# *Trypanosoma brucei* ATR Links DNA Damage Signaling during Antigenic Variation with Regulation of RNA Polymerase I-Transcribed Surface Antigens

**DOI:** 10.1016/j.celrep.2019.12.049

**Published:** 2020-01-21

**Authors:** Jennifer Ann Black, Kathryn Crouch, Leandro Lemgruber, Craig Lapsley, Nicholas Dickens, Luiz R.O. Tosi, Jeremy C. Mottram, Richard McCulloch

**Affiliations:** 1 The Wellcome Centre for Integrative Parasitology, Institute of Infection, Immunity, and Inflammation, University of Glasgow, Sir Graeme Davis Building, 120 University Place, Glasgow G12 8TA, UK; 2 Marine Science Lab, FAU Harbor Branch Oceanographic Institute, 5600 US 1 North, Fort Pierce, FL 34946, USA; 3 Department of Cell and Molecular Biology, Ribeirão Preto Medical School, University of São Paulo, Ribeirão Preto 14049-900 SP, Brazil; 4 Centre for Immunology and Infection, Department of Biology, University of York, Heslington, York YO10 5DD, UK

**Keywords:** *Trypanosoma brucei*, antigenic variation, variant surface glycoprotein, monoallelic expression, RNA polymerase I, protein kinase, ATR, DNA damage signaling, DNA replication stress, immune evasion

## Abstract

*Trypanosoma brucei* evades mammalian immunity by using recombination to switch its surface-expressed variant surface glycoprotein (VSG), while ensuring that only one of many subtelomeric multigene VSG expression sites are transcribed at a time. DNA repair activities have been implicated in the catalysis of VSG switching by recombination, not transcriptional control. How VSG switching is signaled to guide the appropriate reaction or to integrate switching into parasite growth is unknown. Here, we show that the loss of ATR, a DNA damage-signaling protein kinase, is lethal, causing nuclear genome instability and increased VSG switching through VSG-localized damage. Furthermore, ATR loss leads to the increased transcription of silent VSG expression sites and expression of mixed VSGs on the cell surface, effects that are associated with the altered localization of RNA polymerase I and VEX1. This work shows that ATR acts in antigenic variation both through DNA damage signaling and surface antigen expression control.

## Highlights

• Loss of the repair protein kinase ATR in *Trypanosoma brucei* is lethal
• Loss of *T. brucei* ATR alters VSG coat expression needed for immune evasion
• Monoallelic RNA polymerase I VSG expression is undermined by ATR loss
• ATR loss leads to expression of subtelomeric VSGs, indicative of recombination

## Graphical Abstract

**Figure:**
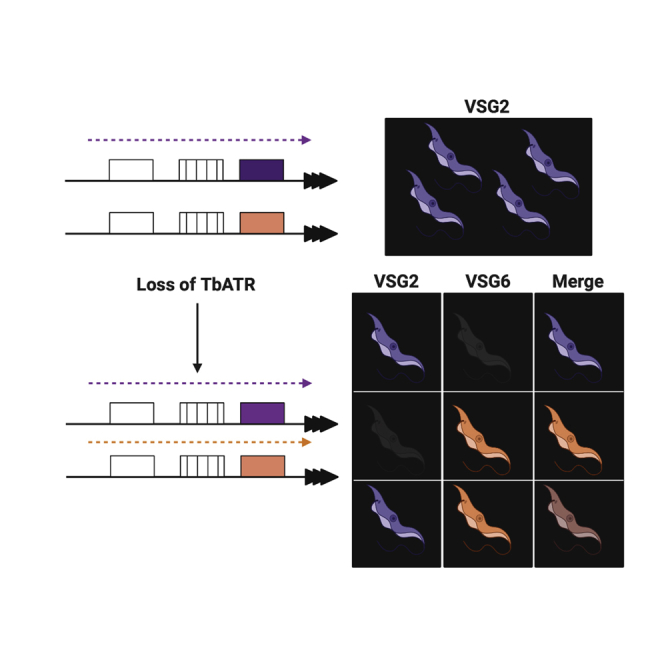

## Teaser

Black et al. show that the loss of ATR, a DNA damage-signaling kinase, is lethal to African trypanosomes and has two effects on surface VSG antigen expression: loss of controls ensuring that only one VSG is transcribed at once, and increased DNA damage that leads to the recombination of normally silent VSGs.

## Introduction

Multiple reactions have evolved to tackle the wide range of stresses faced by cells, including lesions afflicting the genome. A key, early step in genome repair is recognition and signaling of DNA lesions, in which phosphatidylinositol 3-kinase-related kinases (PIKKs) play a central role. Three DNA damage-sensing PIKKs have been described: the DNA-dependent protein kinase catalytic subunit (DNA-PKcs), ataxia-telangiectasia mutated (ATM), and ataxia telangiectasia and Rad3-related (ATR) kinases. Each PIKK is recruited to damaged DNA by distinct binding partners, providing recruitment to specific lesions and activation of specific repair pathways (Lovejoy and Cortez, 2009). DNA-PKcs and ATM are both recruited to DNA double-strand breaks (DSBs), but while the former is targeted to the lesion by the Ku heterodimer and directs non-homologous end-joining repair, the latter is recruited by the Mre11-Rad50-Xrs2 complex and directs homologous recombination (HR). ATR is recruited to single-stranded DNA coated by replication protein A (RPA) through its interaction with ATRIP (ATR interacting protein) (Wang et al., 2017) and, in mammals, ETAA1 (Zou, 2017). Activation of ATR-ATRIP requires further recruitment of TopBP1 and the Rad9-Hus1-Rad1 complex. Single-stranded DNA forms in many settings, meaning that ATR has been implicated in the repair of DSBs and intra-strand crosslinks (Sirbu and Cortez, 2013) and in telomere homeostasis (Maciejowski and de Lange, 2017). However, damage signaling by ATR is the most intimately linked with replication stress, in which it stabilizes replication forks that encounter impediments to their passage, such as damage, DNA secondary structures, the transcription machinery, and RNA-DNA hybrids (Zeman and Cimprich, 2014, Saldivar et al., 2017). The role of ATR in the replication stress response is to limit replication fork collapse, allowing the stalled replisome to resume DNA synthesis, and involves the regulation of cell-cycle progression, coordinating the usage of sites of DNA replication initiation (called origins), and the modulation of replisome activity.

*Trypanosoma brucei* is one of several causative agents of African trypanosomiasis, afflicting both humans and livestock (Morrison et al., 2016). All salivarian trypanosomes are extracellular parasites and avoid elimination by the mammalian adaptive immune response via stochastic changes in their variant surface glycoprotein (VSG) coat. Such surface antigen switching (antigenic variation) is widespread among pathogens, but it has evolved remarkable mechanistic complexity in *T. brucei*. In any given cell, only a single *VSG* is normally actively transcribed, generating a homogeneous VSG coat (Manna et al., 2014). VSG transcription occurs in telomeric bloodstream VSG expression sites (BESs), of which ∼15 are present (Berriman et al., 2002, Hertz-Fowler et al., 2008). The single active BES is transcribed by RNA polymerase I (Pol I) and localizes to an extranucleolar body (the expression site body [ESB]) in the *T. brucei* nucleus (López-Farfán et al., 2014, Navarro and Gull, 2001). Perturbation of a number of processes undermines BES monoallelic expression, including telomere (Jehi et al., 2014a, Jehi et al., 2016, Yang et al., 2009) and nuclear envelope integrity (DuBois et al., 2012, Maishman et al., 2016), chromatin status (Hughes et al., 2007, Povelones et al., 2012, Denninger et al., 2010, Narayanan and Rudenko, 2013, Alsford and Horn, 2012, Aresta-Branco et al., 2016), chromatid cohesion (Landeira et al., 2009), and inositol phosphate signaling (Cestari and Stuart, 2015). In addition, potentially kinetoplastid-specific monoallelic control factors are present, such as VEX1 (Glover et al., 2016), which acts with more widely conserved chromatin-associated factors (Faria et al., 2019). Trypanosomes can undergo an apparently coordinated process (Chaves et al., 1999), in which the single actively transcribed BES is changed, but how this reaction is executed (Figueiredo et al., 2008), initiated (Batram et al., 2014), and signaled (see below) has been less studied.

A further route for VSG switching is the recombination of a silent VSG into the BES (McCulloch et al., 2015), using a genomic archive numbering >2,000 VSGs and pseudogenes (Berriman et al., 2005, Cross et al., 2014, Müller et al., 2018). Extensive evidence indicates that HR, catalyzed by RAD51 (McCulloch and Barry, 1999) and mediated by further factors (Hartley and McCulloch, 2008, Trenaman et al., 2013, Dobson et al., 2011, Proudfoot and McCulloch, 2005, Devlin et al., 2016, Kim and Cross, 2010, Kim and Cross, 2011), directs the switching of functionally intact *VSG*s. It is less clear how VSG pseudogenes are recombined, but the combinatorial assortment of these sequences generates huge levels of expressed VSG diversity in chronic infections (Marcello and Barry, 2007, Hall et al., 2013, Mugnier et al., 2015, McCulloch and Field, 2015, Jayaraman et al., 2019). As for transcriptional switching, the trigger for VSG recombination is still being sought, with BES DSBs (Boothroyd et al., 2009, Glover et al., 2013a), BES replication (Devlin et al., 2016, Devlin et al., 2017, Benmerzouga et al., 2013), telomere shortening (Hovel-Miner et al., 2012), and RNA-DNA hybrids (Briggs et al., 2018, Nanavaty et al., 2017) having been suggested.

Understanding how VSG switching is signaled will bring us closer to revealing the nature of the reaction trigger(s), including the DNA lesion(s) that may direct VSG recombination, and may address whether switching is linked to genome replication and how the reaction integrates into the cell cycle. To date, no work has inquired into whether any PIKK contributes to antigenic variation. Here, we show that the loss of *T. brucei* ATR (TbATR) in mammal-infective cells results in rapid growth impairment, heightened sensitivity to a range of DNA-damaging agents, and accumulation of three nuclear markers of DNA damage, which is consistent with an essential role in genome maintenance. In addition, the loss of TbATR leads to the increased expression of silent VSGs from across the archive and undermines BES expression control. These effects are concomitant with the accumulation of γH2A in the active BES, silent BESs, and subtelomeres, as well as with the altered localization of VEX1 and Pol I. Thus, we reveal a mechanistic link between DNA damage signaling, VSG switching, and monoallelic control of VSG expression during *T. brucei* immune evasion.

## Results

### TbATR Is Essential for *T. brucei* Proliferation and for Survival following DNA Damage

A putative homolog of the ATR kinase, TbATR, has previously been identified in *T. brucei* (Parsons et al., 2005), and preliminary RNAi analysis revealed the impaired proliferation of bloodstream form (BSF) *T. brucei* cells (Jones et al., 2014). However, several proteins involved in the mediation of TbATR activity have yet to be identified in *T. brucei*, including ATRIP and the downstream target CHK1 (checkpoint kinase 1) (Goto et al., 2015). A homolog of TopBP1 has been predicted (Genois et al., 2014) but not validated. The 9-1-1 complex plays important, novel roles in *Leishmania* genome maintenance (Damasceno et al., 2013, Damasceno et al., 2016, Damasceno et al., 2018, Nunes et al., 2011), but interaction with TbATR directly or indirectly has not been assessed, and no work has examined 9-1-1 function in *T. brucei*. Thus, how (and if) TbATR acts in damage signaling, including conservation of its associated machinery, is unknown.

**Figure 1:**
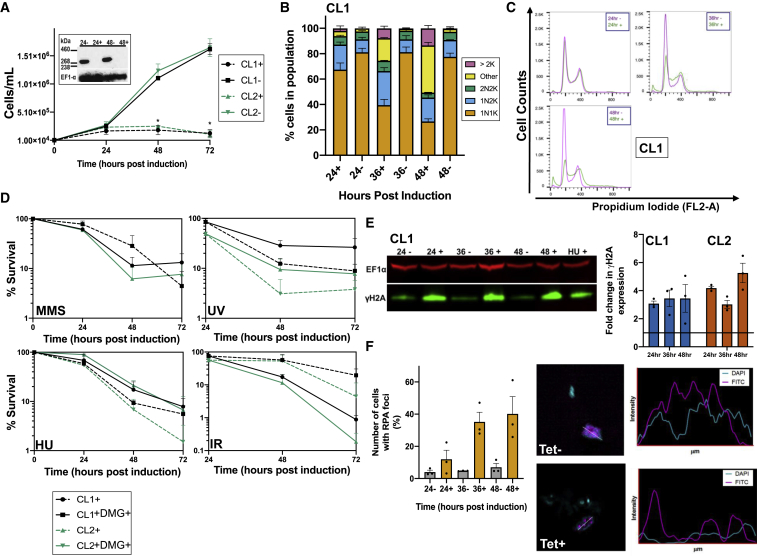
Loss of TbATR Halts *T. brucei* Growth and Increases Nuclear Genome Damage (A) Growth of two clones (CL1, black; CL2, green) after TbATR RNAi induction (+, dashed lines) or when RNAi was not induced (−, solid lines). ± SEM is shown; ^∗^p < 0.05, Mann-Whitney *U* test. Abundance of TbATR^12myc^ in CL2 is shown (insert) after 24 or 48 h of growth with and without RNAi (+ and −, respectively); EF1α acts as a loading control. (B and C) Cell-cycle progression after RNAi monitored by DAPI staining (B) and flow cytometry (C). For DAPI, the number of cells ± SEM in each stage is displayed as a percentage of the total population; >200 cells counted per experiment. For flow cytometry, graphs depict the mode number of cells. (D) Survival of RNAi-induced (+) cells is shown as a proportion (± SEM) of uninduced cells over time in the absence (solid line) or presence (dashed line) of DNA damage (DMG) caused by methylmethanosulfonate (MMS; 0.0003%), UV radiation (UV; 1,500 J/m^2^), hydroxyurea (HU; 0.06 mM), and ionizing radiation (IR; 150 Gy); data are shown for CL1 (black lines) and CL2 (green lines). (E) Expression of γH2A (green) after 24, 36, or 48 h of growth with (+) and without (−) RNAi; EF1α (red) serves as a loading control, and levels are compared with 48 h of growth of uninduced cells in the presence (+) of 0.06 mM HU. The graph shows fold-change (± SEM) in the levels of γH2A in clones CL1 and CL2 after 24, 36, or 48 h of growth with RNAi relative to uninduced cells (set at 1) after normalization using the EF1α signal. (F) Quantification of the percentage (± SEM) of cells in the population that harbor RPA2-myc foci after 24, 36, or 48 h of growth with (+) and without (−) RNAi; >200 cells counted per experiment. Representative images of Tet− and Tet+ cells harboring RPA2-myc foci (magenta) are shown alongside an intensity plot of signal localization; DNA was DAPI stained (cyan).

To examine the effect of TbATR loss, *in vitro* proliferation of BSF cells after tetracycline (Tet)-induced RNAi was examined in two clones, one expressing the kinase from its own locus translationally fused to 12 copies of the myc epitope at the C terminus (TbATR^12myc^). In both clones, growth ceased from 24 h (Figure 1A). qRT-PCR of both clones (Figure S1A) and western analysis of the TbATR^12myc^-expressing clone (Figure 1A) showed that growth impairment was accompanied by reduced levels of TbATR RNA and the loss of detectable myc-tagged protein from 24 h after RNAi induction. The loss of TbATR compromised cell-cycle progression as revealed by the evaluation of DNA content through DAPI staining (Figures 1B, S1B, and S1C) and flow cytometry (Figure 1B, quantification in Figure S1D). Accumulation of cells in S/G2 phase was observed from as early as 24 h post-RNAi induction. In addition, cells harboring aberrant nuclear and kinetoplast DNA configurations were observed in the population from 36 h (∼30%–40% at 48 h post-induction), and up to 10% of the population at 48 h lacked detectable nuclear DNA (“zoids”). To ask whether TbATR plays a role in the DNA damage response, we examined whether its loss sensitizes BSF cells to genotoxic stress by evaluating parasite survival during growth (Figure 1D) in the presence of methyl methanesulfonate (MMS, an alkylator) or hydroxyurea (HU, a ribonucleotide reductase inhibitor), or after exposure to ionizing radiation or UV (a nucleic acid cross-linker). Relative to uninduced controls, cell survival after the loss of TbATR was markedly reduced following exposure to UV and growth in HU. In addition, survival was impaired at late stages of growth in the presence of MMS, which is consistent with a previous study (Stortz et al., 2017). Survival after exposure to ionizing radiation improved after the loss of TbATR. These data indicate that the loss of TbATR compromises BSF proliferation and sensitizes cells to a number of genotoxic agents, suggesting that PIKK contributes to the response of *T. brucei* to a variety of induced DNA lesions.

### Loss of TbATR Leads to Increased Nuclear DNA Damage

To ask whether the above phenotypes reflect nuclear roles for TbATR, we tested whether RNAi causes discernible genome damage. The phosphorylation of histone H2A on Thr130 has been described in *T. brucei* (Glover and Horn, 2012, Devlin et al., 2016) and in *Leishmania major* (Damasceno et al., 2016) after exposure to different genotoxic stresses or repair gene mutation, and thus represents a kinetoplastid variant of the γH2A(X) damage-response nuclear chromatin modification (Biterge and Schneider, 2014). In uninduced TbATR RNAi cells, anti-γH2A antiserum recognized some protein in western blots, and these levels increased from 24 h after TbATR RNAi (Figure 1E). Immunofluorescence (IF) with anti-γH2A antiserum showed that the changes in protein level seen in western blots after TbATR loss were reflected in both increased numbers of cells with γH2A nuclear signal and increased nuclear signal intensity (data not shown). To probe the nuclear damage further, we examined the localization of RPA and RAD51. RPA is a heterotrimer that binds single-stranded DNA and is phosphorylated by ATR during replication stress in other eukaryotes (Vassin et al., 2009). RPA subunit 2 (RPA2) was endogenously myc tagged at its C terminus (Glover et al., 2019) in the TbATR RNAi CL1 cell line and its location determined by indirect IF. In the absence of damage, RPA2-myc mainly localized diffusely throughout the nucleus (Figure 1F, top panel; Figure S1E, top panel), although a small proportion of cells (<10%) harbored a more intense focus or foci (Glover et al., 2019). After growth in the presence of HU or MMS, more cells could be detected with RPA2-myc foci (Figure S1E). The same effect was seen after the loss of TbATR (Figures 1F and S1F); from 24 h post-RNAi, there was a pronounced increase in the number of cells with RPA2-myc foci, reaching ∼40% of the population at 48 h. RAD51 has previously been shown to relocalize into discrete foci in the *T. brucei* nucleus upon the induction of a DNA DSB or after treatment with DNA-damaging agents (Proudfoot and McCulloch, 2005, Dobson et al., 2011, Hartley and McCulloch, 2008, Trenaman et al., 2013, Devlin et al., 2016, Glover et al., 2008). Consistent with these studies, ∼1% of uninduced RNAi cells harbored detectable nuclear foci, with the majority of anti-RAD51 signal in IF seen diffusely across the nucleus and cytoplasm (Figure S1G). However, 36 and 48 h post-RNAi, the number of cells with detectable RAD51 subnuclear foci increased (to ∼7% of the population; Figure S1G). The increased levels of γH2A and the focal accumulation of RPA and RAD51 indicate that nuclear DNA damage arises following the loss of TbATR. The increased levels of γH2A and RPA foci appeared to precede the accumulation of RAD51 foci, perhaps indicating that the latter structures form following the generation of single-stranded DNA and the deposition of variant histone on the damage caused by TbATR loss.

### Altered VSG Transcription Emerges Rapidly after TbATR RNAi

**Figure 2:**
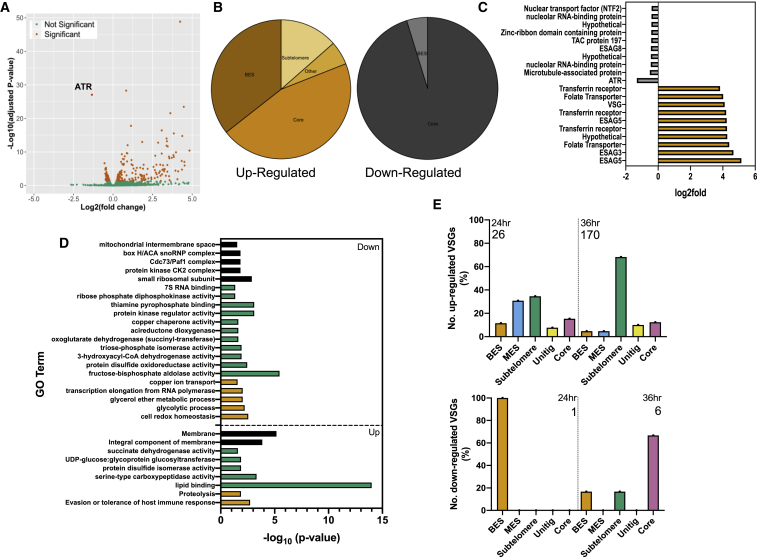
ATR RNAi Leads to Derepression of Surface Antigen Gene Expression in Bloodstream Form *T. brucei* (A) A volcano plot showing differentially expressed transcripts 24 h after RNAi relative to uninduced controls. Log10-adjusted p values for each gene are plotted against the log2 transformed fold-change; data are averages from three biological replicates and transcripts are annotated as follows: significant change in abundance (orange), non-significant (green), and ATR (red). (B) Pie charts summarizing differentially expressed transcripts (left, increased; right, decreased) 24 h after RNAi; the number of genes in each category is expressed as a percentage of the total gene number, and genes were categorized based on their genomic location (core genome, BES, subtelomere, and unmapped unitigs). (C) Top 10 differentially increased (orange) or decreased (gray) transcripts following RNAi. (D) Summary of GO terms significantly enriched in the differentially expressed gene cohort relative to the expected number of genes in the genome. Enriched GO terms in the up- or downregulated cohorts are depicted as −log_10_ (p value) and categorized as biological process (yellow), cell location (black), and molecular process (green; see Table S1). (E) Graphs show the percentage of the total number (indicated) of significantly up-or downregulated VSGs found in BES, MES, subtelomere, unitig, or core locations 24 and 36 h post-RNAi.

To ask whether TbATR loss results in altered gene expression, total RNA was prepared, in triplicate, after 24 h of RNAi induction and subjected to RNA sequencing (RNA-seq), comparing changes in gene-specific read abundance relative to uninduced cells (Figure 2; Figure S2 shows quality control of the RNA-seq). To map sequence reads not only to the core genome but also to VSG-containing subtelomeres of the Lister 427 genome (including the BES) (Müller et al., 2018), MapQ filtering was applied (Hutchinson et al., 2016). A total of 289 transcripts (including TbATR) were significantly differentially expressed (p < 0.005) in the RNAi-induced cells relative to the uninduced (Figure 2A; Table S1). Slightly more genes (56%) were found to increase in transcript abundance than to decrease (Figure S2D). However, ∼50% of the genes that increased in RNA abundance were subtelomeric, compared with only 6 subtelomeric (4%) genes among the cohort of 126 downregulated genes (Figure 2B). Moreover, the extent of RNA level increases was uniformly greater than the extent of decreases (Figure 2C shows the top 10 genes in each cohort). These data suggest that the loss of TbATR has the most rapid and pronounced effect on reducing transcriptional silencing of *T. brucei* subtelomeric genes, which we explored further by examining the genes affected. Gene Ontology (GO) term enrichment analysis (Figure 2D; Table S1) revealed that the most pronounced changes (in terms of number of genes affected and level of enrichment) were in the upregulated cohort, most notably in functions associated with evasion or tolerance of host immune response (biological process), membrane (cellular location), and lipid binding (molecular process). Consistent with this, 35% of the total number of significantly increased reads corresponded to genes from the silent BESs (Figure 2B); moreover, these genes showed the greatest increases (Table S1; 0.87–5.1 log_2_fold). Also prominently represented (∼13%; 0.25–4.1 log_2_fold increases) were VSGs and pseudogenes from outside the BES that mapped to the subtelomeres. Notably, only 6 BES-localized genes displayed reduced expression, 5 of which were located in the active VSG expression site (BES1; Table S1, −0.10 to −0.44 log_2_fold). In the downregulated cohort, a wider range of predicted gene functions was seen (Figure 2D).

RNA-seq at this timepoint after TbATR RNAi does not clearly reveal the potential pathways of core gene expression changes that may reveal the functions regulated by TbATR to enact its putative signaling functions. Instead, these data indicate that the earliest and most pronounced effect of TbATR loss is altered transcription of genes within the BESs, as well as further, non-BES VSGs.

### TbATR RNAi Leads to Loss of Monoallelic VSG Expression and Increased Expression of Select VSGs from Throughout the Silent Archive

Given that the above RNA-seq analysis suggested that TbATR RNAi has a pronounced impact on the expression of BES and subtelomeric VSGs, we sought to investigate this further. We performed RNA-seq after 36 h of RNAi (as before, in triplicate using the two RNAi clones; Figures S2A–S2C for RNA-seq quality control). To explore these data, we evaluated the genomic locations of VSG transcripts found to have significant increases or decreases in abundance (Figure 2E; Table S2). A total of 26 VSG transcripts were upregulated 24 h after RNAi, whereas at 36 h, 170 VSG RNAs increased significantly. The relative distribution of these VSGs changed from 24 to 36 h. At 24 hr post-induction, 42% of upregulated VSGs were either located in a BES or a metacyclic VSG expression site (MES), whereas 37% were located in the subtelomeric arrays (including both intact and pseudogenes). In contrast, 65% of upregulated VSGs were subtelomeric array genes or pseudogenes after 36 h of RNAi, with BES and MES VSGs now representing 10% of the total. A small number of upregulated VSGs at each time point have to date only been mapped to unitigs, so their location in the genome is uncertain. At 24 h, the only downregulated VSG transcript was found in the active BES (BES1; VSG2), although at 36 h, one other, subtelomeric VSG showed a reduced level of transcript. These data suggest that the earliest effects of TbATR loss are not limited to the transcription of BES VSGs, but that increased expression of VSGs is also seen from the MESs that are normally transcriptionally silent in BSF cells (Graham et al., 1999). In addition, the activation of VSGs that are not resident in either a BES or an MES increases with time after RNAi, and such activation is not limited to intact genes. In both the upregulated and downregulated gene cohorts, examples of predicted VSGs were also found that are located within the core of the genome (Figure 2F). These genes almost certainly encode poorly understood VSG-related proteins (Marcello and Barry, 2007), which are not subject to monoallelic transcription and are not exclusively transcribed in BSF cells. Since it is unknown which Pol transcribes the VSG-related genes, and because TbATR RNAi caused both modest increased and decreased RNA levels, it is unclear how these effects may relate to the larger number of differentially expressed VSGs known to be involved in antigenic variation.

**Figure 3:**
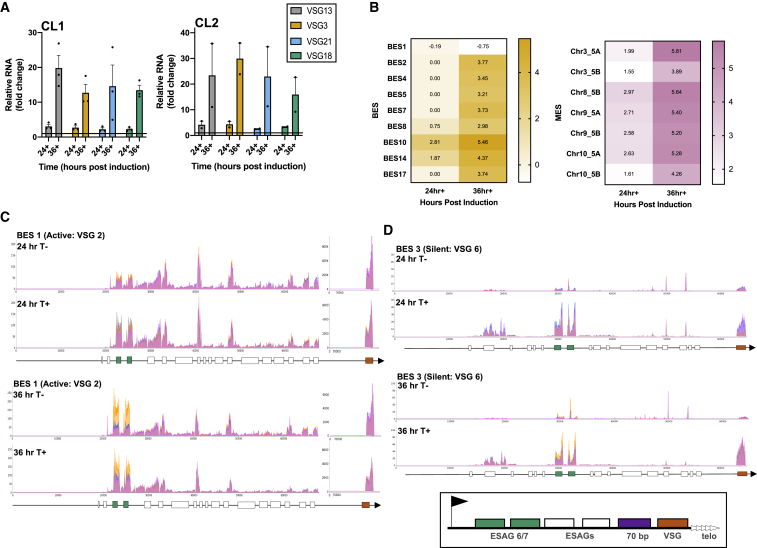
Loss of TbATR Impairs Control of VSG Expression Site Transcription (A) qRT-PCR of VSGs within 4 silent BES are shown (24 and 36 h post-RNAi, +) as fold-change in level relative to uninduced cells (−; set at 1); data are shown for clones CL1 and CL2, and error bars denote ± SEM. (B) Heatmaps of differentially expressed BES and MES VSG transcripts, plotted as log_2_fold change in +RNAi relative to −RNAi. (C and D) RNA-seq read depth across the active BES (BES1; C) and one silent BES (BES3; D) after 24 and 36 h of growth with (T+) or without (T−) RNAi; data from three replicates are overlaid (pink, blue, and orange). ESAG6 and ESAG7 genes are shown in green, other ESAGs in white, and VSG in orange. The boxed graphic shows a simplified layout of a BES (telo, telomere; arrow, promoter).

To check the RNA-seq, we performed RT-qPCR of four silent BES VSGs and confirmed significantly increased levels of each in RNAi-induced cells relative to controls after 36 h of growth (Figure 3A). We also performed RT-qPCR to examine the levels of VSG2 transcript (Figure S3A), but we could find only limited evidence for a decrease. However, RT-qPCR of this gene may be confounded by the very high abundance of this transcript (Tables S1 and S2), and reduced VSG2 RNA levels are consistent with BES-specific mapping and accumulation of cells lacking a VSG2 protein coat (see below). Comparing the log_2_fold change in RNA-seq read depth of all predicted VSGs in BESs and MESs (Figure 3B) showed that VSG transcript levels from all of the silent loci increased from 24 to 36 h after RNAi. Given this finding and the RNA-seq description of significantly reduced levels of four expression site-associated gene (ESAG) transcripts from BES1, allied to significant increases in ESAG transcripts from several silent BESs at 24 h (Table S1), we mapped the data from both 24 and 36 h using MapQ filtering to all BESs (Figures 3C, 3D, and S3B) and MESs (Figure S3C). The mapping revealed a number of things.

First, read mapping indicated that reduced levels of transcript in active BES1 after TbATR RNAi were not limited to VSG2, since reduced read depth was also seen across the telomere-proximal ESAGs (Figure 3C). This effect did not, however, extend across the BES; no such loss was apparent for the two genes encoding the *T. brucei* transferrin receptors ESAG6 and ESAG7 (Steverding et al., 1994), which are immediately downstream of the promoter and were the only significantly upregulated transcripts from BES1 (Table S1). Second, when examining the silent BES, it was apparent that levels of increased gene-specific reads were not uniform across transcription units but, instead, were most pronounced proximal to the promoter and telomere (Figures 3D and S3B). Telomere-proximal increases after TbATR RNAi appeared to only represent the VSGs, and increased ESAG expression was mainly accounted for by the increased abundance of ESAG6 and ESAG7 transcripts, as well as increased levels of transcripts for folate transporters (Figure S3B) encoded by ESAG10 and found downstream of a duplicated BES promoter at some telomeres (Hutchinson et al., 2016, Gottesdiener, 1994). Third, examining the MES revealed that the loss of TbATR caused highly specific increased read mapping to the VSGs, with no associated increase or decrease in upstream genes (Figure S3C). Since the MESs do not contain ESAGs and the upstream genes are Pol II transcribed, these data indicate that TbATR RNAi at 24 h most strongly affects Pol I-transcribed genes.

Finally, the RNA-seq data were mapped to the subtelomeres and VSG-containing unitigs (Figure S3D), confirming that some of the VSGs in these loci are activated by the loss of TbATR, and showing that in some cases, increased reads after TbATR loss map to only part of the predicted gene.

### TbATR RNAi Leads to Changes in VSG Coat Composition

**Figure 4:**
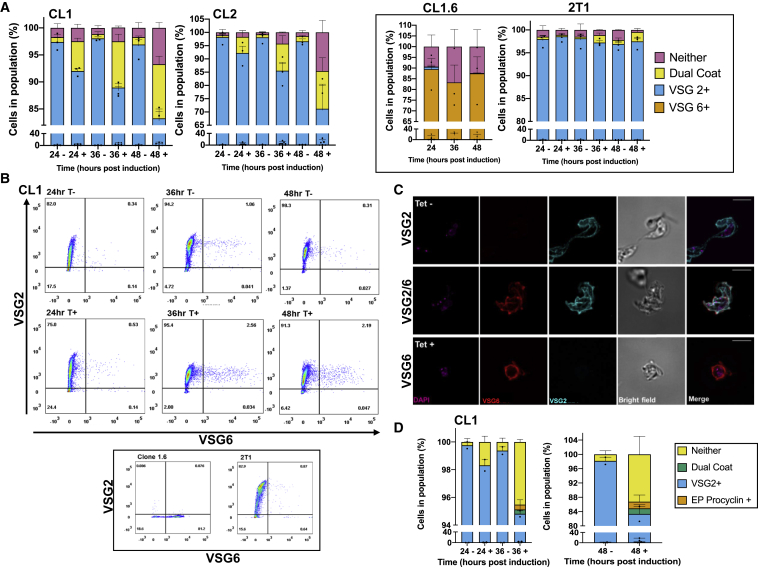
Loss of ATR Results in Changes in VSG Coat Expression (A) VSG2 and VSG6 expression by immunofluorescence 24, 36, and 48 h after RNAi induction (+) in CL1 and CL2, or without induction (−). Individual cells were scored for the presence of just one VSG (VSG2^+^, cyan; VSG6^+^, red), both VSGs (dual coat, yellow), or neither VSG (gray); numbers are expressed as a percentage of the total population ± SEM (200 cells counted per time point per experiment). Control cell lines (CL1.6, expressing mainly VSG6, and the 2T1 parental RNAi cell line, expressing mainly VSG2) are shown in the black-outlined box. (B) Analysis of VSG2 and VSG6 expression by flow cytometry after 24, 36, and 48 h of growth with (T+) or without (T-) RNAi; >10,000 events were analyzed per sample and time point. For comparison, 2T1 and CL1.6 cells are shown. (C) Representative images of CL1 cells and + RNAi and −RNAi (Tet), stained with anti-VSG2 and anti-VSG6 antiserum; scale bars, 5 μm. (D) Expression of EP-procyclin and VSG2 24, 36, and 48 h +RNAi (+), or −RNAi (−). Individual cells were scored for the presence of VSG2 or EP-procyclin and quantified as in (B).

To ask whether changes in VSG RNA after TbATR loss extend to VSG protein expression on the cell surface, we next performed IF on unpermeabilized cells, before and after TbATR RNAi, with two antisera, recognizing either VSG2 (active BES1) or VSG6 (silent BES3) and scoring for the expression of the two VSGs on individual cells (Figures 4A–4C and S4A). In conjunction, flow cytometry was used to analyze larger numbers of cells, also distinguishing cells that expressed one, both, or neither of the VSGs (Figures 4B and S4A). Both approaches produced comparable results, as did comparison of the two RNAi clones. In the absence of TbATR RNAi induction, >98% of cells expressed only VSG2, reflecting monoallelic control of BES transcription and being consistent with the parental RNAi cells grown on tetracycline (Figure 4A). RNAi led to a progressive decrease in cells that stained with only anti-VSG2 antiserum (∼80% and ∼70% of cells after 48 h in clones 1 and 2, respectively; Figures 4A–4C and S4A). Concomitantly, there was a progressive increase in cells that either did not stain with antiserum against either VSG (∼5%–15% after 48 h; Figures 4A, 4B, and S4A) or stained with both anti-VSG2 and anti-VSG6 antiserum (∼10%–15% after 48 hr; Figures 4A–4C and S4A). Cells expressing VSG6 but not VSG2 after TbATR RNAi were present, but rare (Figures 4A–4C and S4A). The detection of two VSGs on the cell surface indicates the loss of monoallelic BES expression or delayed coat switching. Cells without VSG2 in the coat indicate that TbATR depletion can also lead to discontinued expression of the active VSG and, presumably, expression of an undetected VSG or VSGs. Both findings are consistent with the RNA-seq data.

The RNA-seq data (Table S1) provided evidence that TbATR RNAi caused changes in the expression of some Pol I-transcribed genes in addition to the VSGs, including transcripts associated with the coat (known as procyclin) exclusively expressed in the insect stage of the parasite (on procyclic form [PCF] cells). Therefore, we performed an IF analysis on unpermeabilized cells, before and after TbATR RNAi, with antisera recognizing VSG2 (active BES1) or EP-procyclin, and scoring for the expression of cells harboring both VSG2 and EP-procyclin on individual cells (Figures 4D, S4B, and S4C). In the absence of TbATR RNAi induction, as seen previously, >98% of cells appeared to exclusively express VSG2. However, at both 36 and 48 h post-induction of TbATR RNAi, a small percentage of cells (<4%) were seen to express a dual VSG2-EP-procyclin coat (Figures 4D, S4B, and S4C). The detection of a dual BSF-PCF surface coat reveals wider transcription alterations in these parasites, and perhaps suggests that TbATR plays a broader role in monitoring or controlling Pol I transcription.

### Loss of TbATR Leads to Altered Localization of VEX1

**Figure 5:**
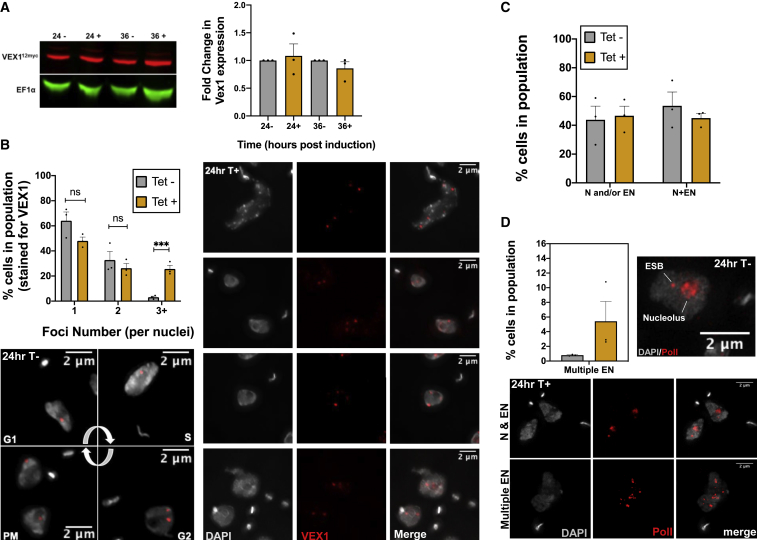
Altered Localization of VEX1 after ATR RNAi (A) Immunoblot of VEX1^−12myc^ (red) expression after 24 or 36 h of growth with (+) and without (−) RNAi; EF1α (green) serves as a loading control. The graph depicts levels of VEX1^−12myc^ protein after normalization using EF1α (set to 1.0). (B) Analysis of VEX1^−12myc^ foci number at 24 h of growth with (Tet+, cyan bars) or without (Tet−, gray bars) RNAi. DNA was stained with DAPI and used to determine the number of individual cells harboring 1, 2, or ≥3 (3+) VEX1^−12myc^ foci. Numbers are expressed as a percentage of the total number of cells counted (± SEM). Images show VEX1^−12myc^ localization (red) after 24 h of growth with (T+) and without (T−) RNAi; DAPI-stained DNA is gray (scale bar, 2 μm). (C) Pol I and ESB after 24 h of growth with (Tet+) and without (Tet−) RNAi. Cells were categorized as having a single subnuclear focus, indicating either nucleolar (N) or extranucleolar staining (EN) staining (N and/or EN), or harboring two clearly distinct foci (N+EN), suggesting both a nucleolus and an ESB; values represent the percentage (± SEM, n = 3) of total cells counted (>100 per experiment). (D) Analysis of the number of cells harboring >2 extranucleolar foci (multiple EN) per single cell 24 h after RNAi (data plotted as in C). Image on the right is a representative example of Pol I (red) in an uninduced cell, while the images below show representative images of Pol I distribution following RNAi (see also Figure S5; DAPI-stained DNA is gray; scale bar, 2 μm).

The BES and VSG expression changes described above after TbATR RNAi display a striking overlap with those seen after RNAi against VEX1 (Glover et al., 2016, Hutchinson et al., 2016), a factor that localizes specifically to the active BES and may be a component of the extranucleolar *T. brucei* ESB. To ask whether the effects of TbATR loss may be mediated through VEX1, we expressed a 12myc-tagged variant of the factor from its own locus (Figure S5A) (Glover et al., 2016) in the TbATR RNAi cells and examined expression and localization, with and without RNAi, using anti-myc antiserum (Figures 5A, 5B, S5B, and S5C). Cells lacking 12myc-tagged VEX1 were used as negative controls, and no staining could be seen (Figure S5B). RNAi-mediated loss of TbATR had no discernible effect on the abundance of VEX1^12myc^ protein (Figure 5A), but it did affect subnuclear localization. In the absence of RNAi induction, ∼60% of cells with a discernible subnuclear anti-myc signal harbored a single focus of VEX1myc, with a smaller number (∼35%) displaying 2 foci, and a very small number (<5%) showing ≥3 VEX1myc foci (Figure 5B). These numbers are largely consistent with previous work (Glover et al., 2016), and DAPI staining confirmed that in virtually all of the cases in which cells had two VEX1myc foci, they were in late stages of S phase or in G2 (Figure 5B). After 24 h of RNAi induction, at the stage at which VSG expression changes were detected, the number of cells with one or two VEX1myc foci appeared to show a modest, although non-significant decrease. A significant increase in cells harboring ≥3 VEX1 foci was seen at the same time (increasing to ∼20% of the population). Examples of such cells are shown in Figures 5B and S5C, where it is notable that aberrant numbers of VEX1myc foci were not limited to S or G2 phase cells. These data reveal that the loss of TbATR in BSF *T. brucei* perturbs the localization of VEX1, resulting in the accumulation (or persistence) of >2 VEX1 foci in a single cell.

### Loss of TbATR Alters Localization of Pol I

Since *VSG* transcription is catalyzed by Pol I sequestered to the actively transcribed BES, we next asked whether the altered VEX1 localization after TbATR RNAi was reflected in changed Pol I localization. To do so, IF with anti-Pol I antiserum (Glover et al., 2016) was compared in cells grown for 24 h with or without TbATR RNAi, the time point at which growth was first impaired and when VSG expression and VEX1 localization changed (Figures 5C, 5D, S5D, and S5E). Cells were first categorized as those displaying only a single subnuclear signal, meaning that we could not see a separate nucleolus and ESB, or as having two distinct foci, indicating nucleolar and extranucleolar signals, the latter of which was most likely the ESB. Consistent with previous reports (Navarro and Gull, 2001, Kerry et al., 2017), ∼55% of uninduced cells showed 2 anti-Pol I signals (Figures 5C and S5D). TbATR RNAi resulted in a modest decrease (from ∼55% to ∼45%) in the numbers of cells lacking two clearly separate Pol I signals (Figures 5C and S5E), despite the increase in number of cells with ≥3VEX1myc foci. A more striking effect of TbATR RNAi was a pronounced increase in the number of cells with >2 anti-Pol I subnuclear foci (Figures 5D and S5E). One explanation for the increased numbers of Pol I foci is that they correspond with the increased numbers of VEX1-12myc foci after the loss of TbATR, suggesting that they represent new ESBs. Alternatively, the change in Pol I foci numbers may represent nucleolar breakdown or mis-segregation. Nonetheless, it seems plausible that the VEX1 and Pol I perturbations are connected.

### TbATR Depleted Cells Accumulate VSG-Localized DNA Damage

**Figure 6:**
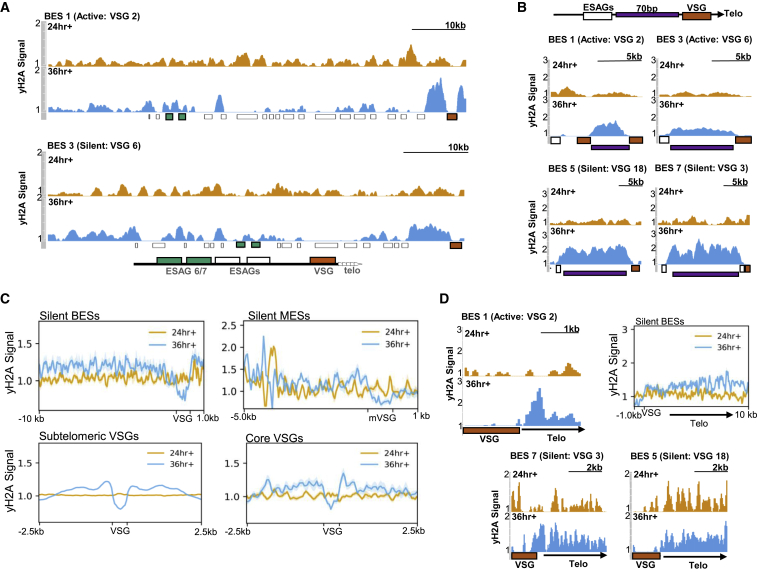
TbATR Loss Results in the Accumulation of VSG-Associated Damage (A) γH2A ChIP-seq enrichment across the active BES (BES1, VSG2) and a silent BES (BES3, VSG6) after 24 (orange) and 36 h (blue) of growth with RNA induction (+). γH2A ChIP-seq signal enrichment (y axis) is shown as a ratio of reads in RNAi-induced samples relative to uninduced samples (each normalized to cognate input sample). VSG is shown as a red box, ESAG6 and ESAG7 as green boxes, and other ESAGs as white boxes. (B) Enrichment of γH2A in RNAi-induced cells relative to uninduced across the 70-bp repeats (purple box) in the active BES1 and in three silent BESs (3, 5, and 7). (C) Metaplots showing γH2A signal enrichment after RNAi across all silent BESs, silent MESs, subtelomeric VSGs, and core VSGs (in each, VSGs are scaled to 500 bp and regions up- and downstream are plotted). (D) γH2A signal enrichment after RNAi in the active BES1 and two silent BES (5 and 7) across the region extending from the end of the VSG (red) to the telomere (arrow, telo); the inset shows a metaplot of γH2A signal for all of the silent BESs across the same region.

TbATR depletion results in increased γH2A levels. However, the location of this damage in the nuclear genome is unknown. To address this, we performed chromatin immunoprecipitation followed by next-generation sequencing (ChIP-seq) using anti-γH2A antiserum. Samples were collected from 1 clone after 24 and 36 h of growth, with and without RNAi induction, and the reads mapped to the Lister 427 genome (Müller et al., 2018) using MapQ filtering. Here, we focus this ChIP-seq analysis on VSGs (Figures 6 and S6). Enrichment of γH2A was observed in the active BES after TbATR RNAi (Figure 6A, upper plot), but the accumulation of the modified histone was uneven across the transcription unit; signal increased from 24 to 36 h within the 70-bp repeats upstream of the VSG (Figure 6B) and in sequences downstream of the VSG (Figure 6D), but such enrichment was less marked across the ESAGs (Figure 6A). γH2A levels also increased in the silent BES (Figure 6A; lower plot), with similar accumulation from 24 to 36 h on the 70-bp repeats (Figure 6B) and telomere-proximal regions (Figure 6D). Furthermore, γH2A levels also increased around the MES VSGs (Figure 6C), although the extent of γH2A enrichment downstream of the VSGs did not appear as marked as was seen in the BES. Finally, γH2A enrichment was seen around non-BES and non-MES VSGs (Figures 6B and 6C). Pronounced signal enrichment was seen upstream and downstream of VSGs located in the subtelomeres (∼2,000) and in the genome core (27). For all of the VSGs, it was notable that γH2A signal enrichment was mainly found flanking, not within, the predicted VSG or pseudogene coding sequence. These data indicate that the increased levels of γH2A after TbATR RNAi are at least partly due to DNA damage accumulation across the VSG archive.

## Discussion

**Figure 7:**
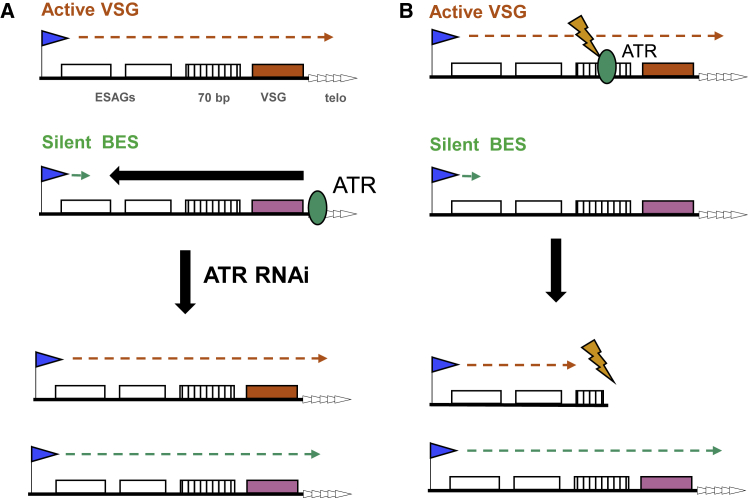
Two Models for ATR Function in *T. brucei* VSG Expression (A) Transcription (green arrow) from the Pol I promoter (arrow) of a silent BES is suppressed (black arrow) by ATR (green circle) and does not traverse the ESAGs (white boxes), 70-bp repeats (hatched box), VSG (pink box), or telomere repeats (arrayed arrowheads). In contrast, TbATR does not impede the transcription (orange arrow) of the single active BES (active VSG, red box). After TbATR RNAi, silencing is compromised, and transcription can extend across the silent BES. (B) TbATR recognizes and signals the repair of lesions (orange lightning bolt) within the actively transcribed VSG BES. The loss of TbATR means that lesions are not effectively repaired and the integrity of the active BES is compromised (e.g., loss of VSG), which is lethal and selects for cells expressing a silent VSG BES.

Cessation of growth after RNAi suggests that TbATR is essential in *T. brucei*, which is consistent with previous analyses (Jones et al., 2014, Stortz et al., 2017, Fernandez-Cortes et al., 2017). Such critical ATR importance is also found in yeast (Cha and Kleckner, 2002) and mammals (Brown and Baltimore, 2000), but it is not universal, since ATR null mutants are viable in plants (Culligan et al., 2004). What TbATR functions are essential for *T. brucei* is unclear. One possibility is TbATR acting in a critical DNA repair pathway, since loss of the PK results in increased sensitivity to damage, most notably that caused by nucleotide depletion (HU) and DNA cross-links (UV). Such roles are also consistent with elevated levels of γH2A and focal accumulation of RPA2 and RAD51 after RNAi, demonstrating that the loss of TbATR results in increased levels of endogenous nuclear genome damage. γH2A expression in *T. brucei* and *L. major* has been shown to increase after induction of a DSB or exposure to MMS, phleomycin, or HU (Glover and Horn, 2012, Damasceno et al., 2016), indicating that phosphorylation arises due to a range of lesions (Revet et al., 2011, Turinetto and Giachino, 2015). RAD51 and RPA focal accumulation in *T. brucei* has been described after the induction of a DSB (Glover et al., 2008, Glover et al., 2019, Devlin et al., 2016), as well as after treatments that can lead to DSBs (Proudfoot and McCulloch, 2005, Trenaman et al., 2013, Hartley and McCulloch, 2008, Marin et al., 2018). Here, we cannot say what type of nuclear lesion(s) arise after TbATR loss, but the earlier increase in γH2A signal relative to RPA and RAD51 may indicate a link in the formation of single-stranded DNA, perhaps consistent with the same temporal order observed in *T. brucei* PCF cells after ionizing radiation treatment (Marin et al., 2018). Nonetheless, the lack of increased sensitivity of BSF TbATR RNAi cells to ionizing radiation may argue for a distinct role, or for life-cycle differences in the signaling of repair activity (Vieira-da-Rocha et al., 2019). Whether the essentiality of TbATR relates to genome-wide or localized activities will require further work, but γH2A ChIP-seq reveals increased levels of lesions around a wide range of VSGs after TbATR loss, linking the PIKK to antigenic variation. Although such a link may be predicted to relate to conserved roles for ATR in DNA repair, antigenic variation in *T. brucei* relies on two seemingly unconnected reactions: activation of any silent VSG by recombination into the BES and transcription-related reactions that ensure that only one BES is actively transcribed and that a silent BES can be activated as the active site is silenced. Our data implicate TbATR in both reactions (Figure 7).

The events that cause the initiation of VSG switching, by recombination or transcription, are the subject of debate (da Silva et al., 2018, Günzl et al., 2015), as is the cell-cycle timing of switching, although recent data have implicated genome replication as a potentially key event (Devlin et al., 2016, Faria et al., 2019). A number of observations indicate that the loss of TbATR affects all of these processes. First, DAPI and flow cytometry reveal that the loss of TbATR leads to impaired cell-cycle progression, with the accumulation of cells with aberrant DNA content indicating incorrect segregation of the nuclear genome after DNA replication. These effects may relate to roles for ATR in other eukaryotes in recognizing and responding to replication stress (Yazinski and Zou, 2016), including regulating the timing of replication origin firing and activation of dormant origins (Shechter et al., 2004, Chen et al., 2015), stabilization and protection of stalled replication forks (Dungrawala et al., 2015, Hu et al., 2012), and replication at fragile sites (Barlow et al., 2013, Casper et al., 2002). Second, TbATR loss results in the expression of two VSGs on the cell surface, an effect that is allied to altered subnuclear localization of both VEX1 and Pol I, as well as increased RNA of VSGs and ESAGs from silent BESs. These findings may be explained by stalling in the process of transcriptional switching or the loss of monoallelic transcription control. The observation that silent MESs become transcribed after TbATR loss argues for the latter explanation, since the activation of such normally PCF-specific transcription units is also seen after RNAi of nuclear lamina components (DuBois et al., 2012, Maishman et al., 2016), in which deregulation also extends to the procyclin surface coat, as we describe here. Third, RNA-seq reveals that TbATR loss leads to increased RNA levels of subtelomeric array VSGs and pseudogenes and accumulation of γH2A across the VSG archive. Since only a fraction of silent subtelomeric VSGs are activated, and increased reads can arise from only parts of genes, it is unlikely that these effects represent widespread transcriptional VSG deregulation, but instead indicate that the loss of TbATR leads to VSG switching by recombination. These VSG archive-wide effects of TbATR loss are comparable to the mutation of two RNase H enzymes (Briggs et al., 2018, Briggs et al., 2019), although whether this indicates a shared activity on RNA-DNA hybrids is unknown. Equally, why the loss of TbATR results in γH2A accumulation in subtelomeric VSG donors is unclear; perhaps this effect derives from homology searching during VSG recombination (Hicks et al., 2011) or perhaps it reflects off-target effects (Khair et al., 2015) of the unknown machinery that generates lesions in the BES to initiate VSG switching.

How can these disparate effects of TbATR loss on VSG expression be explained? Two scenarios, which may not be mutually exclusive, can be considered (Figure 7). One explanation is that TbATR plays an active role in exerting monoallelic transcriptional control on the BES (Figure 7A). Such a function could occur via TbATR interaction with telomeres, which are protected by the shelterin complex (Feuerhahn et al., 2015), since in other eukaryotes POT1 interacts with ATR to prevent its activation (Denchi and de Lange, 2007), and ATR (and ATM) recruit telomerase (Tong et al., 2015, Moser et al., 2011) and shelterin (Moser et al., 2009) to telomeres. Shelterin binding can cause silencing of subtelomeric genes (Ottaviani et al., 2008), and, in *T. brucei*, RNAi of RAP1 (Yang et al., 2009), TRF (Jehi et al., 2014a), and TIF2 (Jehi et al., 2014b) results in impaired BES silencing or VSG switching, suggesting parallels with the effects of TbATR loss. However, measurements suggest that telomere-directed silencing in *T. brucei* stretches for only a few kilobases (Glover and Horn, 2006) and does not encompass the whole BES. In addition, it seems unlikely that telomere integrity is the basis for such an activity since, in contrast to the rapid BES transcription changes seen after TbATR RNAi, telomere repeat attrition after the mutation of telomerase is slow to accumulate (Dreesen et al., 2005), and excision of the telomere tract in a BES does not elicit a change in BES transcription or switching (Glover et al., 2007, Glover et al., 2013a). Given these limitations, might TbATR act at the telomere through VEX1 and/or its wider interacting partners (Glover et al., 2016, Faria et al., 2019)? There are striking parallels in the effects seen after RNAi depletion of TbATR and VEX1; the loss of either factor results in the increased abundance of silent BESs and MES VSG RNAs, decreased expression of VSG2 from the active BES, and co-expression of at least two VSGs on the cell surface. Moreover, RNA-seq mapping to the silent BESs shows the same pattern of increased transcripts after TbATR and VEX1 RNAi (Hutchinson et al., 2016): increases in promoter- and telomere-proximal gene-specific reads, but little evidence for changed levels of ESAGs centrally located in the BES. In only one way do the available data for TbATR and VEX1 diverge: an increased abundance of procyclin was seen in our data, but was observed only after VEX1 overexpression, not RNAi (Glover et al., 2016). This difference may reflect wider roles for TbATR than VEX1 in genome maintenance. We have no evidence that TbATR interacts with or modifies VEX1, but recent work has revealed that VEX1 interacts with the telomere-proximal features of the active and silent BESs (Faria et al., 2019), and it is striking that we see accumulation of γH2A after TbATR loss in the same region of the transcription units. Nonetheless, the effects of TbATR loss may be indirect. For instance, TbATR RNAi may impair nucleolar integrity (Kidiyoor et al., 2016) or it may respond to DNA damage that impedes Pol I transcription (Larsen and Stucki, 2016). Either function may explain increased Pol I foci after TbATR RNAi, leading to increased VEX1 foci. Although such a connection appears consistent with observations that the inhibition of Pol I transcription leads to apparently concurrent breakdown of the nucleolus and loss of extranucleolar Pol I and VEX1 signal (Kerry et al., 2017), it is at odds with evidence for the ESB being a discrete subnuclear structure (Navarro and Gull, 2001). Thus, given the alterations we see in VEX1 localization after TbATR RNAi, it will be valuable to determine whether TbATR and VEX1 act together to influence the deposition or activity of related factors at the BES and MES.

A different explanation for the effects we describe is that TbATR acts to signal the repair of DNA lesions in the active BES (Figure 7B), which is consistent with the increased levels of BES-localized γH2A after RNAi. Pathways that could initiate VSG switching have been variously suggested as the direct generation of a DSB in the BES (Boothroyd et al., 2009, Glover et al., 2013a), telomere fragility resulting in subtelomeric DNA breaks (Hovel-Miner et al., 2012, Jehi et al., 2014b), damage arising from early DNA replication of the active BES (Devlin et al., 2016, Devlin et al., 2017), and RNA-DNA hybrids (Briggs et al., 2018). TbATR could conceivably recognize and signal any such lesion, and therefore RNAi would lead to the observed reduction in telomere-proximal transcripts in the active BES, since unrepaired lesions could lead to the loss of such sequences because of a failure to halt cell-cycle progression to allow repair. Moreover, the apparent increasing loss of gene-specific RNAs with greater proximity to the telomere may indicate that there is no single site of lesion generation, but instead increasing damage from promoter to telomere, consistent with the greater abundance of γH2A around both the 70-bp repeats and the telomere-proximal region of the BES. In this regard, the established role of ATR in tackling transcription-replication clashes is intriguing (Hamperl et al., 2017), since putative lesion density would follow the direction of BES transcription. In fact, further observations may be consistent with TbATR loss undermining ES integrity due to impaired signaling of replication-associated lesions. First, the actively transcribed BES is replicated earlier in S phase than the silent BES (Devlin et al., 2016). Thus, if damage in the active BES was not signaled by TbATR, leading to switching or loss of monoallelic expression (or both), the timing of BES replication may break down, with greater than a single BES replicated early in S phase, causing each to be bound by VEX1. Chromosome mis-segregation due to the loss of TbATR may have a similar effect, resulting in more than a single VEX1 focus in divided cells. Second, a recent study has shown that the loss of the minichromosome maintenance complex-binding protein (MCM-BP) affects *T. brucei* DNA replication and causes loss of monoallelic VSG expression in a very similar manner to that described here: increased silent BES transcripts from telomere-proximal VSGs and promoter-proximal ESAGs (Kim, 2019). However, the localization of VEX1 was not assessed after MCM-BP RNAi, so it may be premature to compare the two studies.

Monoallelic BES expression leading to a single VSG coat on the surface of a BSF *T. brucei* cell is central to the success of immune evasion by antigenic variation, with parallel processes used in many pathogens (Obado et al., 2016, Glover et al., 2013b, Guizetti and Scherf, 2013). Precisely how a single BES is selectively transcribed while the remaining ∼15 are largely transcriptionally silent is still being unraveled (Günzl et al., 2015). To date, DNA repair factors have not been strongly implicated in monoallelic expression, but instead in VSG switching by recombination. However, it should be noted that mutation of the HR factors RAD51 (McCulloch and Barry, 1999), BRCA2 (Hartley and McCulloch, 2008), and RAD51-3 (Proudfoot and McCulloch, 2005) suppresses the levels of antigenic variation, not only by impairing VSG recombination but also by lowering the levels of transcriptional switching between BESs. In addition, exposure of BSF *T. brucei* to DNA-damaging agents can increase silent BES transcription (Sheader et al., 2004). This work on TbATR lends further evidence to a closer than anticipated link between BES transcriptional control and the DNA damage response, which may explain previously described events in which transcriptional VSG switching and deletion of the active BES occur in concert (Cross et al., 1998, Rudenko et al., 1998). Characterizing the nature of the lesions TbATR acts upon, the signaling targets of TbATR, and the roles of all ESB-associated factors will test these possibilities.

## STAR★Methods

### Key Resources Table

**Table:** 

REAGENT or RESOURCE	SOURCE	IDENTIFIER
**Antibodies**		

Anti-RAD51	McCulloch Lab (University of Glasgow)	Diagnostics Scotland (U.K)
Anti-yH2A	McCulloch Lab (University of Glasgow)	N/A
Anti-VSG2	McCulloch Lab (University of Glasgow)	N/A
Anti-VSG6	Horn Lab (University of Dundee)	Referred to here: https://doi.org/10.1073/pnas.1600344113
Anti-Myc Tag Antibody, clone 4A6	Sigma-Aldrich	Cat#05-724
Anti-EF1α Antibody, clone CBP-KK1	Sigma-Aldrich	Cat#05-235
Anti-EP Procyclic FITC Conjugate	Cederlane	Cat#CLP001F
Anti-Polymerase I	Horn Lab (University of Dundee	Referred to here: https://doi.org/10.1073/pnas.1600344113
Anti-KMX1	Hammarton Lab (University of Glasgow)	N/A
Anti-myc Alexa Fluor® 488 conjugate	Sigma-Aldrich	Cat#16-224
Alexa Fluor® 594 anti-mouse	ThermoFisher	Cat#A-11005
Alexa Fluor® 594 anti-rabbit	ThermoFisher	Cat#A-11012
Alexa Fluor® 594 anti-rat	ThermoFisher	Cat#A-11007
Alexa Fluor® 488 anti-rabbit	ThermoFisher	Cat#A27034
Alexa Fluor® 488 anti-mouse	ThermoFisher	Cat#A28175
IRDye 680RD anti-mouse	Li-Cor	Cat#926-68070
IRDye 800CW anti-rabbit	Li-Cor	Cat#926-32211
Anti-mouse HRP conjugate	ThermoFisher	Cat#62-6520
Anti-rabbit HRP conjugate	ThermoFisher	Cat#65-6120

**Bacterial and Virus Strains**		

MAX Efficiency ™ DH5-alpha Competent Cells	ThermoFisher	Cat#18258012
DH5-alpha Competent Cells	In house prep from above	N/A

**Chemicals, Peptides, and Recombinant Proteins**		

Methyl Methanesulfonate	Sigma-Aldrich	Cat#129925
Hydroxyurea	Sigma-Aldrich	Cat#H8627
DAPI Fluoromount-G®	Southern-Biotech	Cat#0100-20
Propidium Iodide	Sigma-Aldrich	Cat#P4170
Chameleon® Duo Pre-stained Protein ladder	Li-Cor	Cat#928-60000
HiMark Pre-stained Protein Standard	ThermoFisher	Cat#LC5699
Agencourt AMPure XP beads	Beckman Coulter	Cat#A63882
SYBR® Green PCR Master Mix	Applied Biosystems	Cat#4309155

**Critical Commercial Assays**		

TruSeq ChIP Library Preparation Kit	Illumina	Cat#IP-202-1012
ChIP-IT® Express Enzymatic Shearing Kit	Active Motif	Cat#53035
RNeasy Mini Kit (250)	QIAGEN	Cat#74106
DNeasy Blood & Tissue Kit (250)	QIAGEN	Cat#69506
TruSeq Stranded Total RNA Kit	Illumina	Cat#20020596

**Deposited Data**		

Sequence data is deposited in the European Nucleotide Archive	This Paper	Accession Number: PRJEB23973

**Experimental Models: Cell Lines**		

*Trypanosoma brucei:* Lister 427 Bloodstream form	Laboratory of Prof Richard McCulloch	N/A
*Trypanosoma brucei:* TREU 927 Procyclic form	Laboratory of Prof Harry D Koning	N/A

**Oligonucleotides**		

See Table S2 for primer details	N/A	N/A
TbRPA2 Primers FW and RV	Glover et al., 2019	https://doi.org/10.1128/mBio.01252-19
TbVEX1 Primers FW and RV	Glover et al., 2016	https://doi.org/10.1073/pnas.1600344113

**Recombinant DNA**		

pNAT^12xmyc^	Alsford and Horn, 2008	https://doi.org/10.1016/j.molbiopara.2008.05.006
pGL2084 (RNAi parental plasmid: Gateway Adapted)	Jones et al., 2014	https://doi.org/10.1371/journal.ppat.1003886

**Software and Algorithms**		

GraphPad Prism v8.	GraphPad	https://www.graphpad.com
Galaxy Server	Afgan et al., 2018	usegalaxy.org
DeepTools	Ramírez et al., 2014	https://doi.org/10.1093/nar/gkw257
FlowJo v.10	FlowJo	https://www.flowjo.com
Fiji (ImageJ)	Schindelin et al., 2012	https://doi.org/10.1038/nmeth.2019
Bowtie2	Langmead and Salzberg, 2012	http://bowtie-bio.sourceforge.net/bowtie2/index.shtml
HiSat 2	Kim et al., 2015, Pertea et al., 2016	https://ccb.jhu.edu/software/hisat2/index.shtml
RStudio		https://www.rstudio.com/
SamTools	Li et al., 2009	http://samtools.sourceforge.net
IMARIS v8.2	Oxford Instruments	https://imaris.oxinst.com
DEseq2	Love et al., 2014	https://doi.org/10.1186/s13059-014-0550-8
TopGO	Alexa et al., 2006	https://bioconductor.org/packages/release/bioc/html/topGO.html

**Other**		

Sequence data was aligned to the *T. brucei* Lister 427 HGAP v.10 genome	Version 10 kindly provided by R. Cosentino	Original genome reference (v.9.) http://www.nature.com/articles/s41586-018-0619-8

### Lead Contact and Materials Availability

Further information and reagent/ resource requests should be directed to and will be fulfilled by the Lead Contact, Richard McCulloch (richard.mcculloch@glasgow.ac.uk). This study did not generate unique reagents.

### Experimental Model and Subject Details

2T1 cells (Alsford et al., 2005) were used as a background for RNAi based studies. *T. brucei brucei* Lister 427 cells (Müller et al., 2018) were used for all other BSF based studies. *T. brucei brucei* 927 PCF cells were a kind gift from G.D.Campagnaro (deKoning Lab; University of Glasgow).RNAi inducible cells were grown in HMI-9 medium (GIBCO) supplemented with 20% (v/v) fetal calf serum (low-tet; GIBCO) (Stortz et al., 2017) and RNAi cells were maintained in the 5 μg.mL^-1^ Hygromycin and 5 μg.mL^-1^ Phleomycin. For maintaining myc tagged expressing cell lines, 10 μg.mL^-1^ Blasticidin was added to the media. PCF cells were maintained in SMD79 (Brun and Schönenberger, 1979) supplemented with 10% (v/v) fetal calf serum.

### Method Details

#### Antibody Information

All information regarding antiserum used in this study are detailed in Table S2.

#### Oligonucleotide sequences

Oligonucleotide sequences and raw RT-qPCR data in this study are described in Table S2. Oligonucleotides and plasmids were designed using CLC Genomics Workbench 7 (QIAGEN) or, in the case of RT-qPCR primers, Primer Express® v3.0 (Applied Biosystems) was used. Oligonucleotides were synthesized by Eurofins Genomics (https://www.eurofins.com/). All sequence information was retrieved from TriTrypDB (https://tritrypdb.org/tritrypdb/) and specificity *in silico* confirmed by BLAST (NCBI).

#### Plasmid Design and Cloning

For TbATR RNAi, a construct containing an RNAi target sequence derived from the coding sequence of TbATR was generated using the Gateway cloning strategy as described by Jones et al. (2014). The construct (termed pTL50; kind gift N. Jones) was transformed into the 2T1 parental cell line (kind gift, D. Horn) and two clones were recovered for further analysis (referred to as CL1 and CL2). One allele of TbATR was endogenously tagged at the C terminus with 12 copies of the myc epitope (12myc) using the vector pNATx12myc (kind gift, D. Horn). Cloning was conducted as described in Devlin et al. (2016). Both VEX1 and RPA2 were endogenously tagged using the strategy described above. Both constructs were kindly provided by L. Glover. All genomic DNA was extracted using the Blood and Tissue Extraction Kit (QIAGEN) as per manufacturer’s instructions and stored at 4 ^o^ C until required.

#### RNAi Analysis

Growth curves were performed as described in Stortz et al. (2017). RNAi was induced using 1 μg/ml^-1^ tetracycline. Briefly, cells were seeded at 1 x10^4^ cells.ml^-1^ in 1.2 mLs in a 24 well plate. Cells were counted manually every 24 hr using a Neubauer improved haemocytometer (Marienfeld-Superior, Germany). For those performed in the presence of genotoxic stress, the following concentration or exposure of genotoxic agents were used (unless stated otherwise): MMS (0.0003%), hydroxyurea (0.06 mM), UV (1500 J/m^2^) and IR (150 Gy). For UV and IR, RNAi was induced for 24 hours prior to exposure. For UV exposure, cells were set up in 6 mLs, induced for 24 hr. After 24 hr, cells were transferred to a 6 well dish, placed in a Stratalinker® UV Crosslinker 1800 (Stratagene) without the dish lid and exposed as required. After, the cells were transferred back to a 24 well dish in a volume of 1.2 mLs. For IR exposure, cells were grown in 25 cm^3^ vented flasks in a volume of 5 mLs, induced for 24 hr as described then exposed to a single dose of X-rays. Exposure concentration was determined by length of bombardment time.

#### Cell cycle analysis by DAPI

Cells were seeded at a concentration of 6.25 x10^2^ cells.mL^-1^. Cells were left to grow overnight, the culture divided equally then RNAi induced as stated above. Cultures were then harvested every 24 hr by centrifugation (405 xg for 10 mins). Approximately 2 × 10^6^ cells were collected. The resulting pellet was washed in 1 x PBS, then the cells resuspended in 1x PBS and settled on a Poly-L-lysine (Sigma) treated slide for 5 mins. The supernatant was removed and the cells fixed in 4% formaldehyde for 4 mins. The fixed cells were washed a further 3x in 1 x PBS and DAPI added (DAPI Fluoromount G; Southern Biotech) for 5 mins.

#### Immunofluorescence

For internal antigens including yH2A, RAD51 and myc tagged RPA1, immunofluorescence was performed exactly as described in Stortz et al. (2017). Anti-RAD51 (Diagnostics Scotland, UK) was used at a concentration of 1:1000. To detect RAD51, goat anti-rabbit AlexaFluor 594 was used at a concentration of 1:2000. Cells were permeabilised for 10 mins using 1x PBS/Triton X-100 (Thermo Scientific) for IF of internal myc tagged proteins. Immunofluorescence of surface VSGs was performed as described in Glover et al. (2016) with the following modifications: anti-VSG2 and anti-VSG6 were both used at a concentration of 1:8000; goat anti-rabbit AlexaFluor 488 or goat anti-rat AlexaFluor 594 (Invitrogen) secondary antisera were used at 1:2000. No permeabilization was performed. Staining for EP-Procyclin was performed in the same manner as for VSG staining. EP-Procyclin conjugated to FITC (Cedarlane) was used at a concentration of 1:750. Antibodies were all incubated at room temperature for 1 hr. Immunofluorescence of VEX1^-12myc^ and RNA Pol I was performed as described in Glover et al. (2016) using an antigen retrieval protocol based on urea treatment. Briefly, Antigen Retrievel Buffer (100 mM Tris, 5% (w/v) urea, pH 9.5) was heated to 95 oC in a waterbath. Slides containing parasites (after fixation) were placed in this buffer for 1 minute then immersed in 1x PBS and washed 3x in 1x PBS. Afterward the cells were permeabilsed as described. DAPI staining was performed as above. For KMX-1 staining, anti-KMX-1 amtiserum (kind gift, T. Hammarton) was diluted in 1% BSA only.

#### Immunoblotting

Immunoblotting to assess levels of γH2A, or to detect myc-tagged proteins, was performed and analyzed exactly described in Stortz et al. (2017). Briefly, approximately 2.5 x10^6^ cells were harvested by centrifugation and washed 1x in 1x PBS. The resultant pellet was re-suspended in 1x protein loading buffer (250 μl 4x NuPAGE LDS sample buffer [Invitrogen], 750 μl 1x PBS and 25 μl β-mercaptoethanol). Samples were then boiled immediately for 10 mins at 100°C and stored at – 20°C until required. For C-terminally tagged TbATR, 20 μ 2 x Roche Complete Mini protease inhibitor cocktail (Roche) was added to the extract. SDS-PAGE was used to separate cell lysates on NuPAGE Novex Pre-Cast Gels (ThermoFisher): 4%–12% Bis-Tris, 10% Bis-Tris, 12% Bis-Tris or 3%–8% Tris-acetate gels were used. Gels were run as per the manufacturer’s instructions using either Tris-Acetate running buffer (ThermoFisher) or MOPS (ThermoFisher). Proteins were transferred onto PVDF membrane (Amersham Bio) using a Mini Trans-Blot Cell (Bio-Rad) by electrophoresis (100 V for 2 hr). For TbATR^12myc^ transfer was performed overnight at 400 mA at 4°C. Protein transfer was confirmed by staining with Ponceau-S solution (Sigma). Next, membranes were washed once in 1x PBST (PBS, 0.01% Tween-20 [Sigma]) for 10 mins then incubated for 1 hr (or overnight at 4°C) in blocking solution (1x PBST, 5% Milk powder [Marvel]). The membrane was then washed 1x PBST (10 mins), and incubated in blocking buffer containing the required primary antisera for 1 hr (see Table S2 for antibody concentrations used in this study). Next, the membrane was washed in 1x PBST for 20 mins and incubated with the appropriate secondary antisera for 1 hr .Finally, the membrane was washed in 1x PBST (30 mins) and SuperSignal West Pico Chemiluminescent Substrate (Thermo-Fisher) or ECL Prime Western Blotting Detection Reagent (Amersham) added (incubated for 5 mins). The membrane was stored with either an X-ray film (Kodak) or an ECL Hyperfilm (Amersham) for ∼1 s to overnight and developed using a Kodak M-25-M X-omat processor.

#### Quantification of relative protein levels

Quantification of protein levels was performed as described in Stortz et al. (2017) with the following modifications. Briefly, blots were blocked in 5% milk powder in 1 x PBS overnight at 4°C. Chameleon Duo Pre-Stained Protein Ladder (2 μl; Li-Cor) was loaded to confirm protein sizes. The following secondary antibodies were used: IRDye 680 goat anti-mouse and IRDye 800 goat anti-rabbit (both 1:10,000, Li-Cor). The membrane was washed once in 1x PBST, then again with 1x PBS. Images were captured using an Odyssey CLx Imager (Li-Cor). The band intensity was quantified using the in-built software (ImageStudio). Fold change was calculated in Excel by normalizing each sample to the loading control and calculating the relative fold change to the control sample.

#### RNA preparation and RT-qPCR

To assess gene knockdown using RT-qPCR, RNA was extracted from 1 × 10^7^ BSF *T. brucei* cells using the RNeasy kit (QIAGEN manufacturer’s instructions); three independent extracts were performed for CL1 and two independent extractions were performed for CL2. The samples were stored frozen for less than 1 week at −80 ^o^ C prior to RNA extraction. RNA was treated for 30 minutes at room temperature off column with DNase I (QIAGEN) to minimize DNA contamination. 1 μg of total RNA, quantified using a Nanodrop, was converted to cDNA as per manufacturer’s instructions using Superscript III® Reverse Transcriptase (RT; “First Strand cDNA Synthesis” protocol; Thermo Fischer) using random hexamers. RT minus samples were prepared to control for genomic DNA contamination. All cDNA was stored at −20 ^o^ C until required. The following master mix was set up: 2.5 μl of the appropriate cDNA, 2.5 μl of the appropriate primers (300 nM stock) and 12.5 μl SYBR® Green PCR Master Mix (Applied Biosystems) in a total volume of 25 μl. Samples were set up in a MicroAmp® Optical 96-well Reaction Plates (Thermo Fischer) as triplicates and run in a 7500 Real Time PCR system (Applied Biosystems). The following PCR conditions were used: 50°C for 2 min (x 1), 95°C for 10 min (x 1), 95°C for 15 s followed by 60°C for 1 min (x 40) followed by a dissociation step (95°C for 15 s, 60°C for 1 min, 95 ^o^Cfor 15 s and finally 60°C for 15 s). Amplicon length for each oligonucleotide pair equates to 150 bp. All reactions were set up manually as technical triplicates for each biological replicate and the average CT value for each PCR product calculated. Data was analyzed using the ΔΔCt method (Schmittgen and Livak, 2008) and analysis performed in Excel from the data generated from the PCR run. Actin was used as a reference gene as described previously (Tiengwe et al., 2012).

#### Analysis of VSG expression and cell cycle using flow cytometry

Flow cytometry was performed as described in Glover et al. (2016) to identify VSG6 or VSG2 positive cells: signal from excitation with the BB515 laser (BB515, log) was plotted against the signal from the excitation with the PE-CF594 laser (PE-CF594, log), using as controls 2T1 cells that predominantly express VSG2, and clone 1.6 cells (Glover et al., 2007) that predominantly express VSG6. Approximately 2 × 10^7^ cells were collected for analysis resulting in over 10,000 events collected per sample. Samples were run on a BD Celesta (BD Biosciences) and the data analyzed using FlowJo v.10 (TreeStar). For cell cycle progression, samples were collected as described in Stortz et al. (2017) and were run on a BD FACSCalibur (BD Biosciences) and over 50,000 events captured and analyzed as above. All samples were stored for no longer than one week (prior to antibody staining and analysis) at 4 ^o^ C. RNA was digested with 100 μg.mL^-1^ RNaseA (QIAGEN) for 30 mins at 37 ^o^ C and propidium iodide added to a final concentration of 10 μg.mL^-1^.

#### Imaging and image processing

Images captured on an Axioskop2 (Zeiss) fluorescent microscope used a 63 x DC magnification lens and images were acquired with ZEN software (Zeiss). For images captured on an Olympus IX71 DeltaVision Core System (Applied Precision, GE), a 1.40/100 x lens was used, and images were acquired using SoftWoRx suit 2.0 (Applied Precision, GE). Z stacks were acquired (no more than 10 μm thick) and images de-convolved (conservative ratio; 1024x1024 resolution) using the SoftWoRx software. Super-resolution structural illuminated images were captured on an Elyra PS.1 super resolution microscope (Zeiss), and using images were acquired using ZEN software as Z stacks. Fiji (Schindelin et al., 2012) was used to subtract the background of images and for counting cells. The brightness and contrast for counting were set relative to unstained controls. False colors were assigned to fluorescent channels and the signal enhanced for clear visualization. 3D images were generated using IMARIS software (V8.2; Oxford Instruments) using super-resolution Z stacked images. Scale bars are as stated on images or in legends.

#### RNaseq analysis

Cells were sampled at 24 and 36 hr post RNAi induction, or at equivalent times without induction, from two biological replicates of TbATR CL1 cells and from a single replicate of TbATR CL2, providing triplicate induced and uninduced samples at both time points. In all cases cells were harvested and RNA prepared as previously described (Briggs et al., 2018). Briefly, the RNeasy Mini Kit (QIAGEN) was used to extract total RNA as per the manufacturer’s instructions. Samples were treated with DNase I (QIAGEN) off column for 30 mins. PCR analysis was performed on the isolated RNA to test for the presence of genomic DNA contamination; no contamination could be detected (data not shown). RNA concentration was measured using a Qubit (Thermo Fischer) as per the manufacturer’s instructions prior to library preparation. Extracted RNA was stored at −80 ^o^ C until required. Library preparation was performed after Poly(A) selection using the TruSeq Stranded Total RNA kit (Illumina). Libraries were paired-end sequenced using an Illumina NextSeq 500 and a Mid-Output Flow Cell generating read lengths of 75 bp.

Sequence reads were trimmed (using TrimGalore) and aligned to the T.brucei Lister 427 HGAP v.10 genome (kindly provided by R. Cosentino) (Müller et al., 2018) using HISAT2 (Kim et al., 2015) and the –no-spliced-alignment flag. MAPQ < 1 (SAMTools)(Li et al., 2009); filtering was applied before counting reads mapping uniquely to the coding strand of each gene using htseq-count. Differential expression was performed to compare induced and uninduced samples accounting for differences between the two clones using DEseq2; a FDR ≤ 0.05 was considered significant. The total read count for each sample, the overall variance and the data spread were assessed and found to be comparable. Differentially expressed transcripts were expressed as a log_2_ fold change and plotted against the adjusted p value on the volcano plot. GO term analysis was performed using TopGO using the weight01 algorithm for pruning, with enrichment analysis being performed using Fisher’s exact test.

#### Chromatin immunoprecipitation

Chromatin immunoprecipitation was performed using 3 μg of yH2A monoclonal antiserum (in house produced). Chromatin was prepared exactly as described previously (Briggs et al., 2018) using an adapted protocol of the ChIP-IT® Enzymatic Express Chromatin Immunoprecipitation Kit (Active Motif). Chromatin was sheared using MNase. Library preparation was conducted using the TruSeq ChIP Library Preparation Kit (Illumina). 300 bp fragments were size selected using Agencourt AMPure XP beads (Beckman Coulter). ChIPseq libraries were sequenced on an Illumina NextSeq 500 platform. Trimmed reads (generated using TrimGalore; default settings) were aligned to the Lister 427 HGAP.v10 genome using Bowtie2 (Langmead and Salzberg, 2012). The fold change between IP and input sample read depth was calculated for each sample using DeepTools bamCompare. Library size was normalized by SES(Diaz et al., 2012) and the fold change was expressed as a ratio. The induced sample was normalized to the uninduced sample using bigwigCompare (DeepTools) and expressed as a ratio. Tracks were visualized using IGV and metaplots, and further analysis on normalized ratio files was performed using DeepTools (Ramírez et al., 2014). The majority of the analysis was carried out using the Galaxy Server (Afgan et al., 2018). Gviz package was used to plot ChIPseq signal across genomic regions. Samples were binned into 1000 bp sliding average windows to visualize the data. Circle plots were generated using Circlize from bedgraph data.

### Quantification and Statistical Analysis

#### Data presentation and statistical analysis

Graphical representation of data was performed in Prism 8 (GraphPad) or in RStudio using the following suits: ggplot2, circlize, ggpubr and userfriendlyscience. Statistical analysis was performed in Prism 8 and the appropriate tests conducted are as detailed in the corresponding figure legends. To plot RNaseq mapping across the BES and other regions of interest, reads per base were counted on the forward and reverse strands in the regions of interest. Normalization for read depth coverage was carried out by dividing the per base read counts by a scaling factor comprised of the total read count in that sample divided by 1000000. Plots were generated using MatPlotLib.

#### Graphical Abstract

The graphical abstract was created with BioRender.com

### Data and Code Availability

Sequences used in this study have been deposited in the European Nucleotide Archive. Data can be accessed using the accession number: PRJEB23973.

## Supplemental Information

Document S1. Figures S1–S6

Table S1. 24-h Post-induction RNA-Seq and GO Term Information in BSF *T. brucei* Cells, Related to Figure 2 and STAR Methods RNA-Seq Analysis

Table S2. Oligonucleotides, Reagents, and Figure 1C Raw Values, Related to Figure 2 and STAR Methods qRT-PCR Analysis

Table S3. Raw qRT-PCR Values and Analysis, Related to Figures 3, S1, and S3

Table S4. Raw RNA-Seq Data Relating to VSGs at 36-h Post-TbATR Induction, Related to Figure 2 and STAR Methods RNA-Seq Analysis

Data S1. Additional Plots from the RNA-Seq Mapping, Related to Figures 3 and S3 See Figure S3 for explanation of content.

Document S2. Article plus Supplemental Information
