# Supplementary material for: Trypanosoma brucei ATR Links DNA Damage Signaling during Antigenic Variation with Regulation of RNA Polymerase I-Transcribed Surface Antigens
Source: Cell Rep. 2020 Jan 21;30(3):836–851.e5. doi: 10.1016/j.celrep.2019.12.049 (PMC6988115; doi:10.1016/j.celrep.2019.12.049)
Supplement: Document S1. Figures S1–S6 [file mmc1.pdf]

**Cell Reports, Volume *30***

## Supplemental Information

# *Trypanosoma brucei* ATR Links DNA Damage Signaling during Antigenic Variation with Regulation of RNA Polymerase I-Transcribed Surface Antigens

**Jennifer Ann Black, Kathryn Crouch, Leandro Lemgruber, Craig Lapsley, Nicholas Dickens, Luiz R.O. Tosi, Jeremy C. Mottram, and Richard McCulloch**

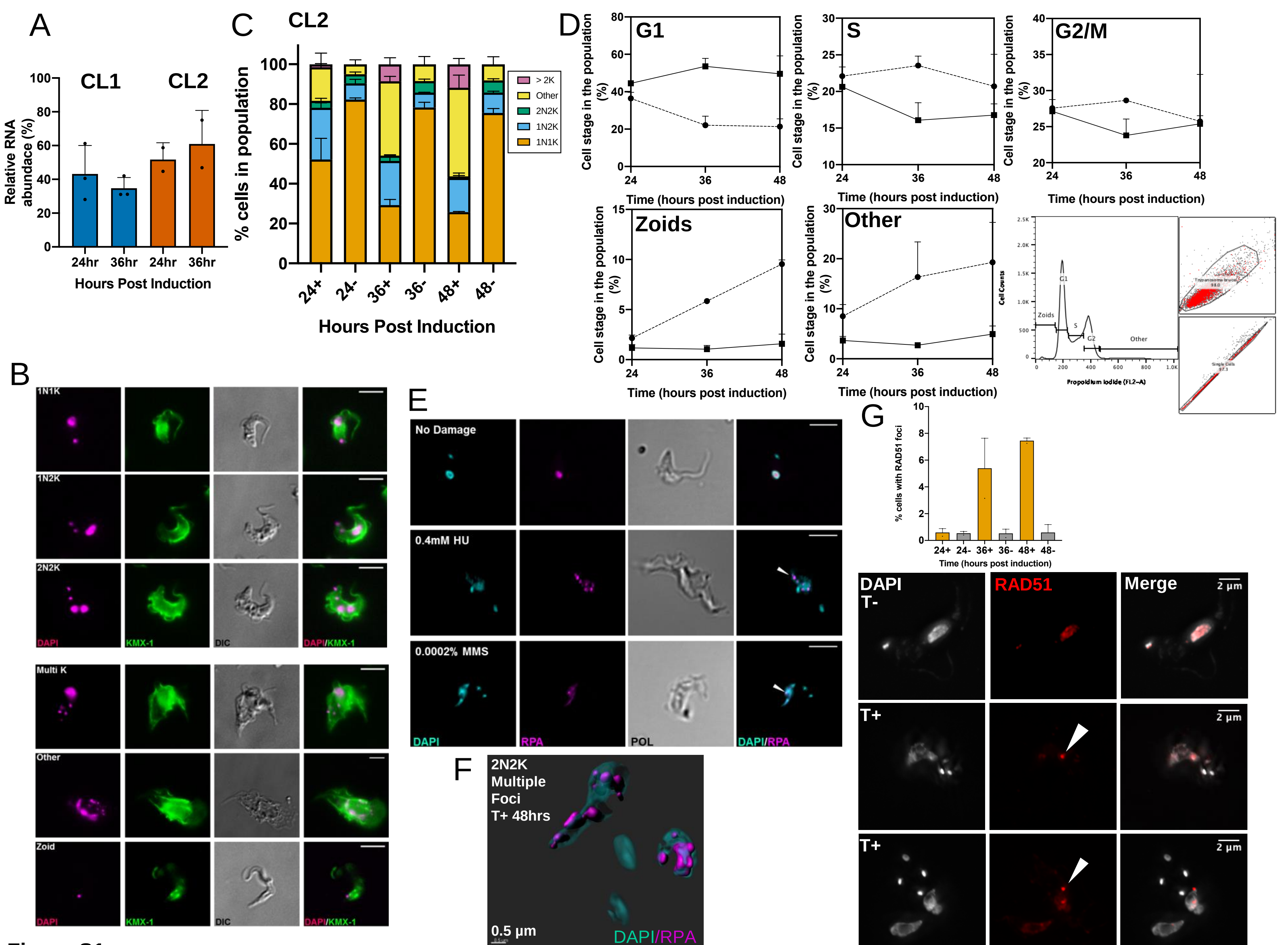
A
CL1
CL2
Relative RNA abundance (%)
24hr 36hr 24hr 36hr
Hours Post Induction
C
CL2
% cells in population
> 2K
Other
2N2K
1N2K
1N1K
24+ 24- 36+ 36- 48+ 48-
Hours Post Induction
D
G1
S
G2/M
Zoids
Other
Cell stage in the population (%)
Time (hours post induction)
Zoids
G1
S
G2
Other
Cell Counts
Propidium Iodide (FL2-A)
B
1N1K
1N2K
2N2K
DAPI
KMX-1
DIC
DAPI/KMX-1
Multi K
Other
Zoid
E
No Damage
0.4mM HU
0.0002% MMS
DAPI
RPA
POL
DAPI/RPA
F
2N2K
Multiple
Foci
T+ 48hrs
0.5 μm
DAPI/RPA
G
% cells with RAD51 foci
24+ 24- 36+ 36- 48+ 48-
Time (hours post induction)
DAPI
T-
RAD51
Merge
2 μm
T+

Figure S1

**Supplementary Figure S1. Effect of RNAi induction against TbATR on BSF *T. brucei* cells. Related to Fig.1.** (A) TbATR RNA abundance assessed by RT-qPCR in two clones (CL1 and CL2) 24 and 36 hrs after RNAi; RNA levels are expressed relative to uninduced cells (set at 100%) at the same times; error bars show ± SEM (n=3 for CL1, and n=2 for CL2). (B) Representative images of uninduced cells (top three panels) and cells induced for 48 hrs with 1 µg.mL$^{-1}$ tetracycline (lower three panels). Cells were stained with DAPI (to visualise the n- and kDNA; magenta) and α KMX-1 antiserum (to visualise tubulin; green). The cell body was visualised by differential interference contrast microscopy (labelled as DIC). Scale bar = 10 µm. Images were captured on an Axioskop 2. (C) Cell cycle progression examined by DAPI staining. The number of cells in each defined cell cycle stage is displayed as a percentage of the total population. Over 200 cells were counted/time point/ experiment. Error bars = ± SEM (n=3). (D) Quantification of cell types before (-) and after (+) RNAi using flow cytometry; graphs depict the percentage of cells in each category relative to the total percentage of cells examined. Error bars = ± SEM (n=2) for CL1. 50,000 events were captured with the number of cells normalised to the mode. DNA was stained with propidium iodide (FL-2A channel). Gating strategy is shown. (E) Representative images of RPA2-myc localisation by indirect immunofluorescence using anti-myc antiserum (magenta) in the absence of induced damage, or after growth in the presence of 0.4 mM HU or 0.0002 % MMS, each for18 hrs. Scale bar = 5 µm. Stacked images were captures on a DeltaVision microscope (Applied Precision). (F) A 3D rendered example of a 2N2K cell harbouring multiple RPA2-myc foci in each nuclei after48 hrs of RNAi. The Image was generated using IMARIS software (V.8.2) from stacked images acquired on an Elyra SR-SIM microscope (Zeiss). Scale bar = 0.5 µm. (G) Cells were collected at 24, 36 and 48 hrs post RNAi induction and RAD51 localisation was performed by indirect immunofluorescence. Cells were counted for the presence RAD51 foci and this number expressed as a percentage of the total number of cells counted. Over 200 cells were counted/time point/ experiment. Error bars = ± SEM (n=2). Representative images of induced (Tet+) and non-induced (Tet-) TbATR RNAi cells at 48hrs. RAD51 foci are marked by white arrows. DNA is stained with DAPI (gray) and RAD51 localised using anti-RAD51 antiserum (red). Stacked images were captured on a DeltaVision microscope (Applied Precision). Scale bar = 2 µm.

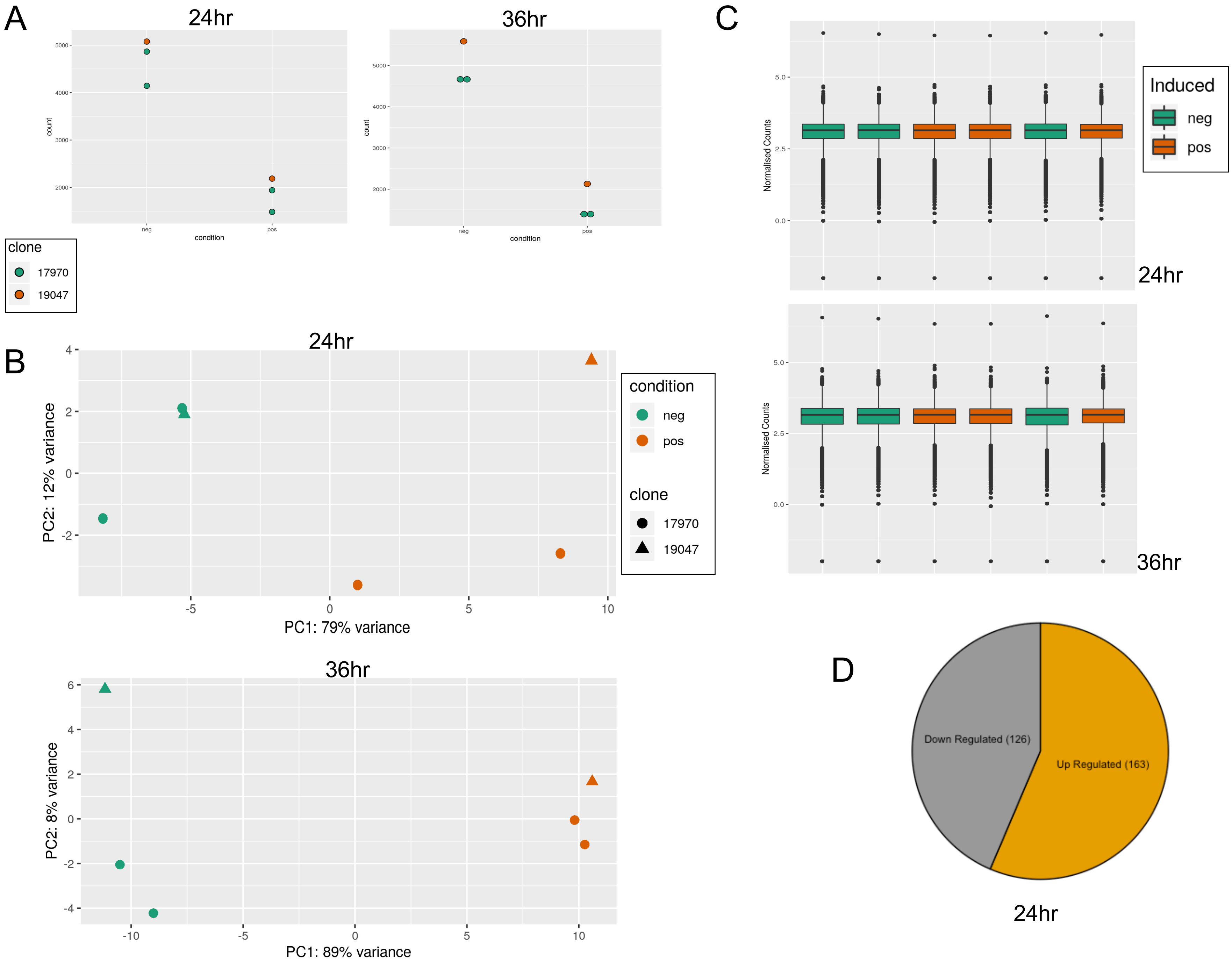
A
24hr
36hr
count
condition
neg
pos
clone
17970
19047
B
24hr
PC1: 79% variance
PC2: 12% variance
condition
neg
pos
clone
17970
19047
36hr
PC1: 89% variance
PC2: 8% variance
C
Normalised Counts
Induced
neg
pos
24hr
36hr
D
Down Regulated (126)
Up Regulated (163)
24hr

**Figure S2**

**Supplementary Figure S2. Quality assessment of the RNAseq data at 24 and 36 hrs post depletion of TbATR by RNAi. Related to Fig.2.** (A) Plot of read counts in each clone separated by condition: CL1 = 19790, green ;and CL2 = 19047, orange. (B) Principle Component Analysis (PCA) was performed on samples from each clone (CL1 = circle, and CL2 = triangle) and in the presence (pos) and absence (neg) of RNAi induction. (C) Boxplot of normalised read counts for each sample in the presence (pos; orange) or absence (neg; green) of RNAi induction. (D) Total number of significantly up- (orange) and down (grey)-regulated transcripts following TbATR depletion by RNAi. Numbers in brackets reflect number of transcripts corresponding to the category.

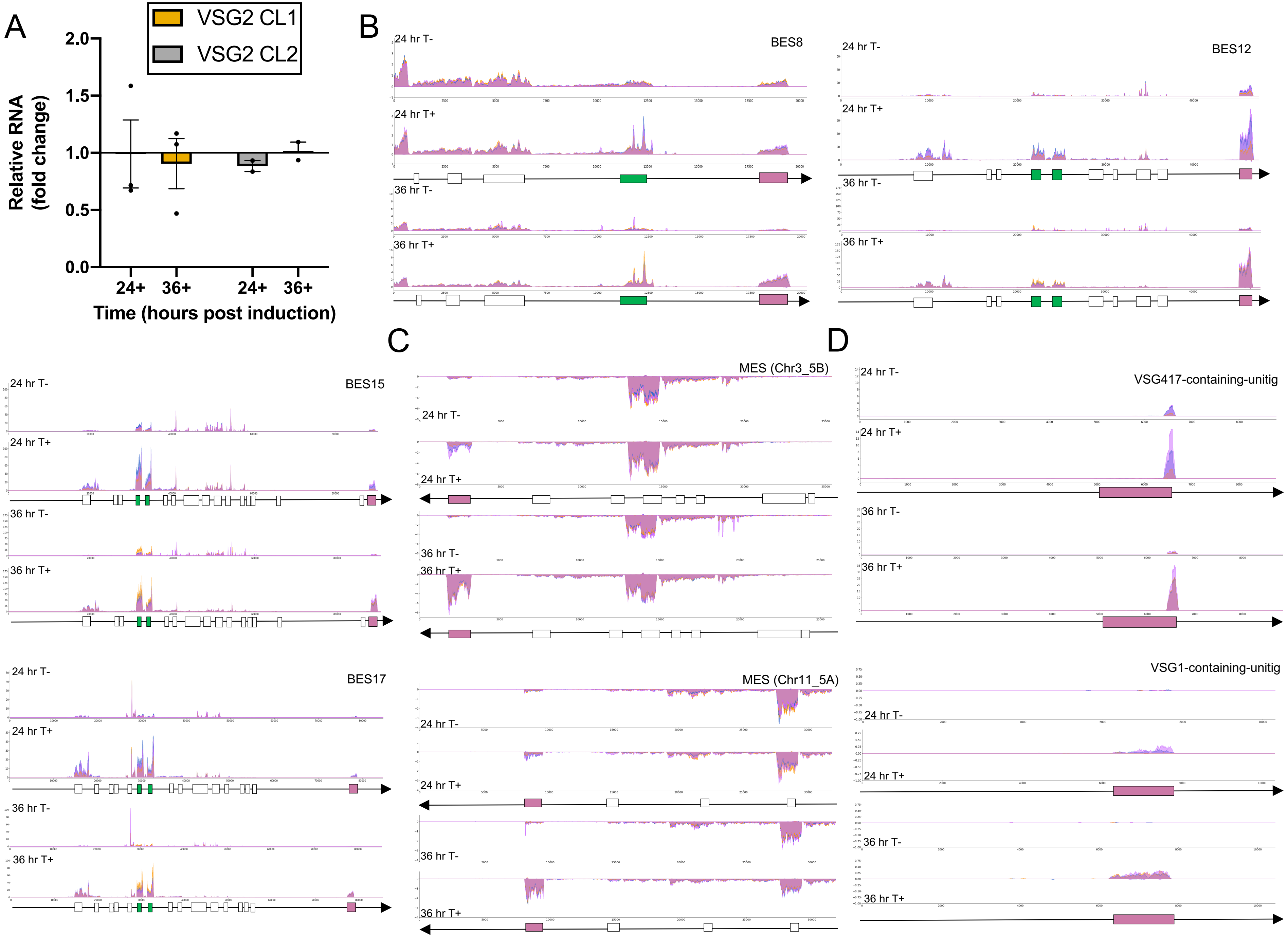
A
VSG2 CL1
VSG2 CL2
Relative RNA (fold change)
2.0
1.5
1.0
0.5
0.0
24+
36+
24+
36+
Time (hours post induction)
B
BES8
BES12
C
MES (Chr3_5B)
MES (Chr11_5A)
D
VSG417-containing-unitig
VSG1-containing-unitig
BES15
BES17
24 hr T-
24 hr T+
36 hr T-
36 hr T+

Figure S3

**Supplementary Figure S3. Loss of TbATR alters VSG transcription in *T. brucei* bloodstream form cells. Related to Fig.3.** (A) RNA levels of VSG2 were assessed by RT-qPCR, at 24 and 36 hrs post RNAi. Levels are shown as fold-change relative to RNA levels uninduced cells (set at 1). Data are shown for two clones (CL1, CL2) and error bars denote ± SEM (n=3 for CL1, n = 2 for CL2). (B) Mapping of MapQ filtered RNAseq reads is shown across four silent VSG bloodstream VSG expression sites (BES) at 24 and 36 hrs growth with (T+) or without (T-) induction of TbATR RNAi; y axes show the number of reads that map relative to location in the transcription units, and data from three replicates are overlaid. ESAG6 and ESAG7 are shown in green, all other ESAGs are shown in white, and the VSG is in pink. (C) Mapping of MapQ filtered RNAseq reads is shown across two metacyclic expression sites (MESs) at 24 and 36 hrs growth with (T+) or without (T-) induction of TbATR RNAi; y axes show the number of reads that map relative to location in the transcription units, and data from three replicates are overlaid. VSG is shown in pink and upstream, non-MES genes are shown in white. (D) Mapping of MapQ filtered RNAseq reads is shown across two unitigs at 24 and 36 hrs growth with (T+) or without (T-) induction of TbATR RNAi; y axes show the number of reads that map relative to location in the transcription units, and data from three replicates are overlaid. The VSG is shown in pink. See Supplemental Item 5 for mapping to further BES, MES, subtelomeres and unitigs.

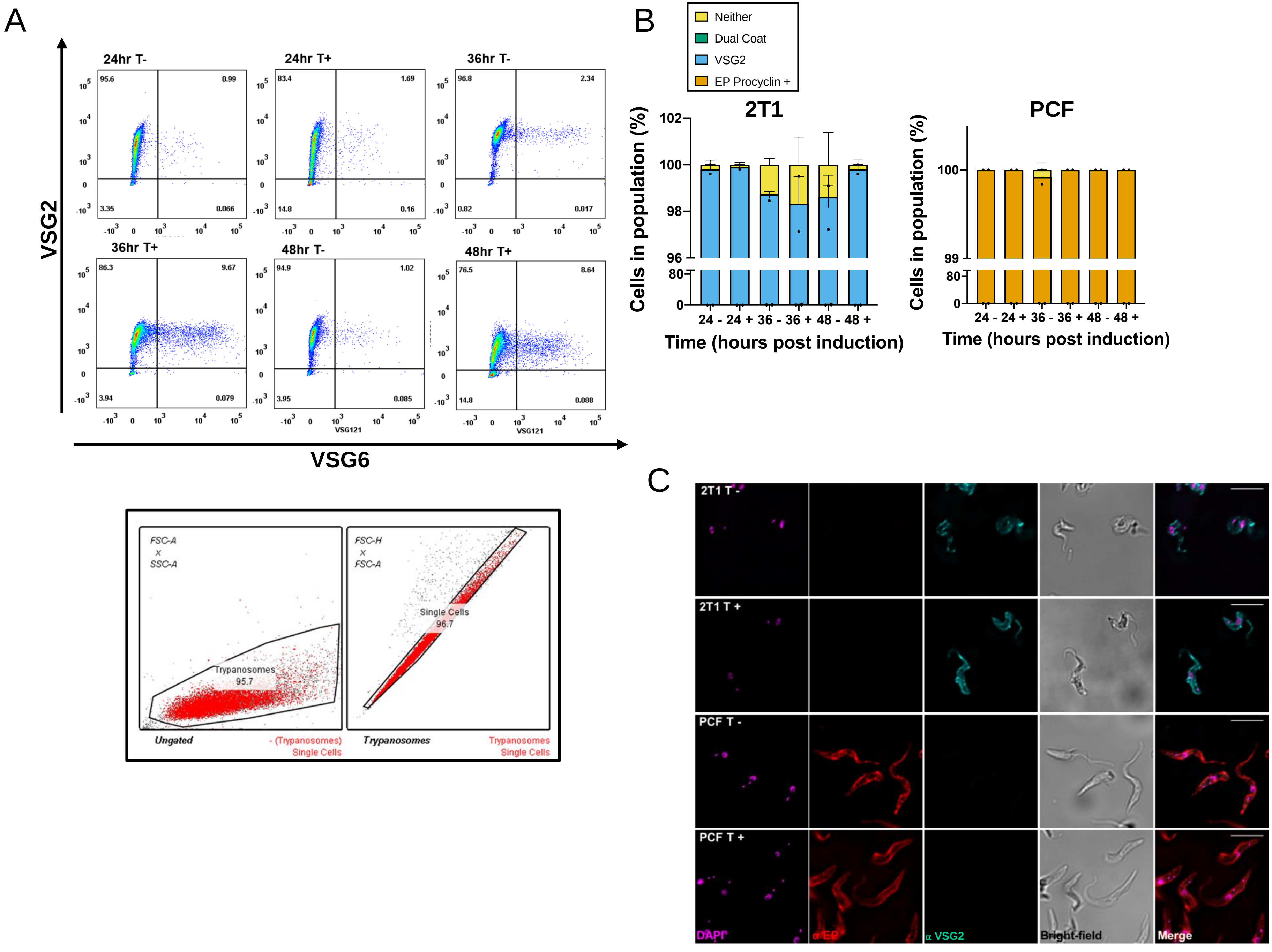
A
24hr T-
24hr T+
36hr T-
36hr T+
48hr T-
48hr T+
95.6
0.99
3.35
0.066
83.4
1.69
14.8
0.16
96.8
2.34
0.82
0.017
86.3
9.67
3.94
0.079
94.9
1.02
3.95
0.085
76.5
8.64
14.8
0.088
VSG121
VSG2
VSG6
FSC-A x SSC-A
Trypanosomes 95.7
Ungated
- (Trypanosomes) Single Cells
FSC-H x FSC-A
Single Cells 96.7
Trypanosomes
Trypanosomes Single Cells
B
Neither
Dual Coat
VSG2
EP Procyclin +
2T1
PCF
Cells in population (%)
24 - 24 + 36 - 36 + 48 - 48 +
Time (hours post induction)
C
2T1 T -
2T1 T +
PCF T -
PCF T +
DAPI
α EP
α VSG2
Bright-field
Merge

**Figure S4**

**Supplementary Figure S4. Loss of ATR in bloodstream form *T. brucei* results in changes in VSG coat expression. Related to Fig.4.** (A) Analysis of VSG expression by flow cytometry. Cells in which TbATR RNAi had been induced (T+) or controls without induction (T-) were collected from CL2 after 24, 36 and 48 hrs growth, stained with anti-VSG2 and anti-VSG6 antiserum and analysed by flow cytometry. Over 10,000 cells were analysed per sample and time point; data from one experiment is shown. The boxed plots detail the gating strategy used in the flow cytometry. To discriminate between healthy and dead cells, side scatter (SSC-A, linear) and forward scatter (FSC-A, linear) were plotted. Cells with high SSC-A signals or very low SSC-A signals suggest these cells are very granular (high) or debris (low). Wide gates were chosen to permit analysis of phenotypic changes following TbATR RNAi. (B) Analysis of VSG and EP-procyclin expression by indirect immunofluorescence. Cells were collected at 24, 36 and 48 hrs after addition or in the absence of tetracycline addition for both control 2T1 and PCF cells. Cells were then stained with anti-VSG2 and anti-EP procyclin antiserum. Individual cells were scored for the presence of VSG, VSG and EP-procyclin, neither, or both surface proteins (dual coat); numbers are expressed as a percentage of the total population and error bars show ± SEM (n=3; >200 cells were counted/time point/experiment. (C) Representative images of 2T1 and PCF cells stained with anti-VSG2 (cyan) and anti-EP-procyclin (red) antiserum; scale bars, 5 µm. N- and K- DNA are stained with DAPI (magenta).

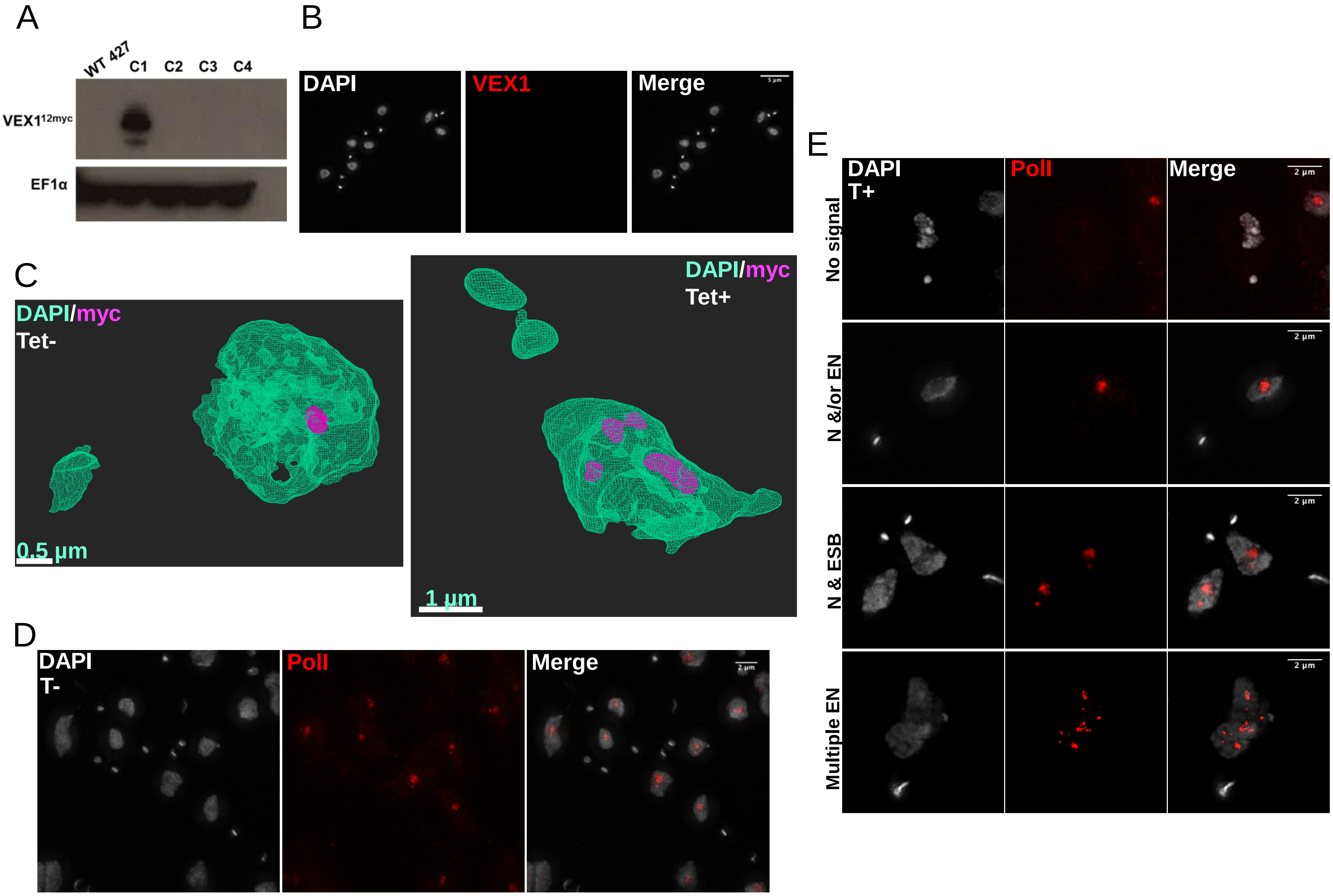
A
WT 427
C1
C2
C3
C4
VEX1^12myc
EF1α
B
DAPI
VEX1
Merge
5 µm
C
DAPI/myc
Tet-
0.5 µm
DAPI/myc
Tet+
1 µm
D
DAPI
T-
PolI
Merge
2 µm
E
DAPI
T+
PolI
Merge
2 µm
No signal
N &/or EN
N & ESB
Multiple EN

Figure S5

**Supplementary Figure S5. Loss of TbATR results in aberrant VEX1 localisation. Related to Fig.5.** (A) Transformant clones putatively endogenously expressing VEX1 tagged with 12 copies of the myc epitope ($VEX1^{-12myc}$) were generated and expression of an appropriately sized protein that reacted with anti-myc antiserum was detected in one (C1); anti-EF1α antiserum was used as a loading control and blotting was performed with wildtype, untagged (WT427) cells. (B) Untagged TbATR RNAi uninduced cells are shown as a control for non-specific anti-myc antiserum binding. N- and K-DNA are DAPI stained (grey) and anti-myc signal (VEX1) would be in red. Images were captured on a DeltaVision microscope; scale bar = 5 µm. No cell outline shown. (C) 3D rendered images of $VEX1^{-12myc}$ localisation before (Tet-) and after (Tet+) RNAi depletion of TbATR were generated using Z-stacked images captured on an Elyra super resolution microscope and complied images using IMARIS software (V.8.2). Scale bars are as stated on the images. N- and K-DNA are stained with DAPI (cyan), and $VEX1^{-12myc}$ detected with anti-myc antiserum (magenta). Cells were collected after 24 hrs growth. (D) Representative images of RNA Pol I localisation without (tet-) induction of RNAi against TbATR in bloodstream form cells. DNA was visualised by DAPI staining (gray), and RNA Pol I detected using anti-Pol I antiserum (red). Images were captured on a DeltaVision microscope; scale bar = 5 µm. (E) Representative images of each category of RNA Pol I localisation after induction of RNAi (Tet+). Scale bar = 2 µm. N &/or EN denotes cells where it was unclear if there was discrete nucleolar and extra nucleolar RNA Pol I signal; N & ESB denotes cells with clearly separate single nucleolar and extranucleolar RNA Pol I signals, indicative of an ESB; no signal indicates cells devoid of any clear RNA Pol I signal; multiple EN denotes cells with greater than 3 nucleolar and extranucleolar RNA Pol I foci.

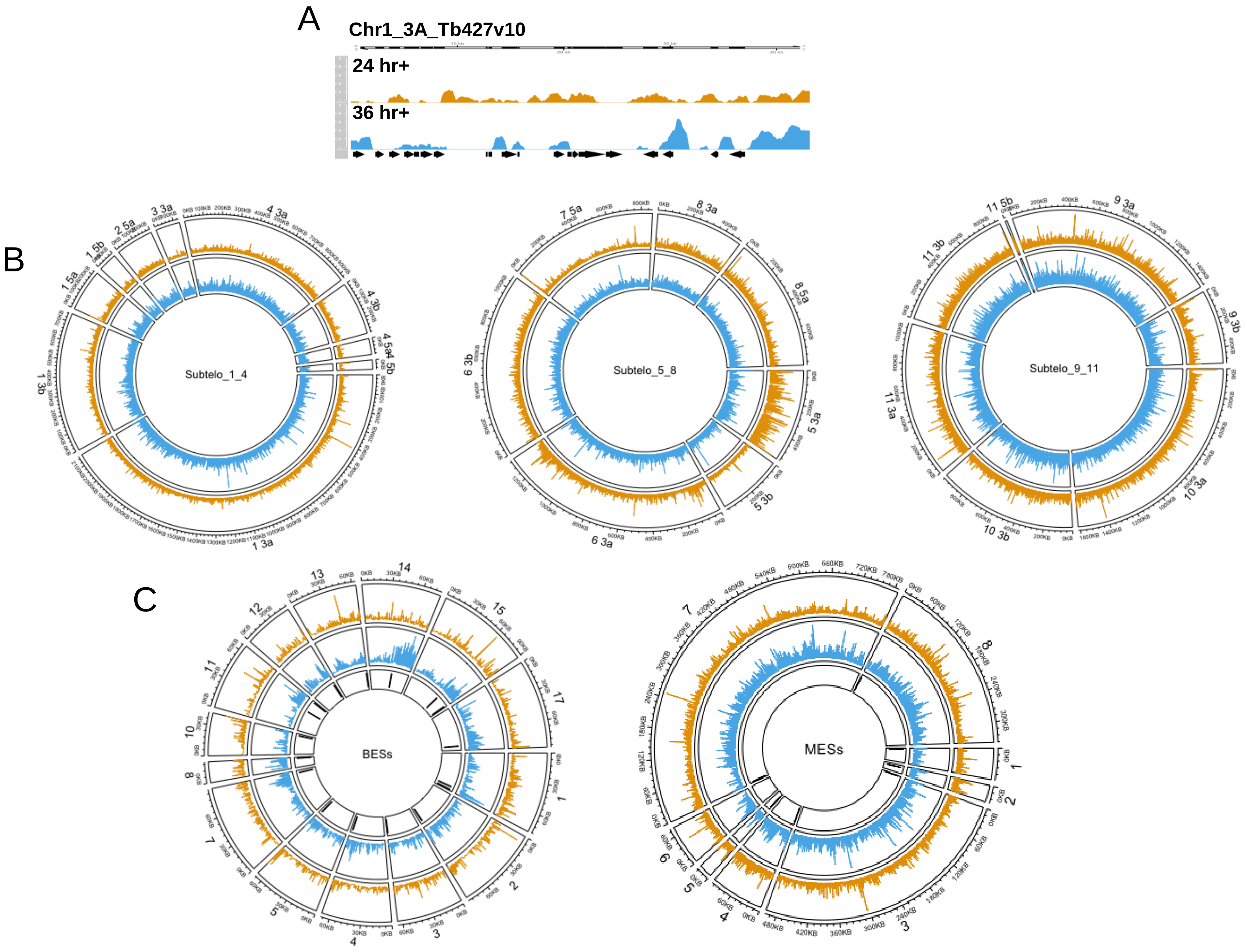
A
Chr1_3A_Tb427v10
24 hr+
36 hr+
B
Subtelo_1_4
Subtelo_5_8
Subtelo_9_11
C
BESs
MESs

**Figure S6**

**Supplementary Figure S6. Loss of TbATR is associated with accumulation of VSG-associated DNA damage. Related to Fig.6.** (A) Plot of yH2A signal across one subtelomeric region of chromosome 1 (as a representative of subtelomeric regions. Data is shown after 24 (yellow) and 36 hrs (blue) growth with RNA induction (+). yH2A ChIP-seq signal enrichment (y axis) is shown as a ratio of reads in RNAi induced samples relative to uninduced (each first normalised to the cognate input sample. Genes are annotated as black arrows (indicating the direction of transcription) (B) Circos plots of enriched regions (> 1.2 fold) from the samples described above are shown across all subtelomeres of the 11 megabase chromosomes of T. brucei Lister 427, with subtelomeric regions in chromosomes 1-4, 5-8 and 9-11 plotted separately to aid visualisation. (C) Circos plots of enriched regions (> 1.2 fold) from the samples described above are shown across all the mapped BESs and MESs of T. brucei Lister 427. Boxes represent the position of the VSG in the ESs. MESs are named as follows: 1 (Chr10_5A), 2 (Chr11_5A), 3 (Chr3_5A), 4 (Chr9_5B), 5 (Chr3_5B), 6 (Chr10_5B), 7 (Chr8_5B) and 8 (Chr9_5A), and plots include upstream genes that are not part of the VSG MES units.
