# Supplementary figures and images for: Trypanosoma brucei ATR Links DNA Damage Signaling during Antigenic Variation with Regulation of RNA Polymerase I-Transcribed Surface Antigens
Source: Cell Rep. 2020 Jan 21;30(3):836–851.e5. doi: 10.1016/j.celrep.2019.12.049 (PMC6988115; doi:10.1016/j.celrep.2019.12.049)

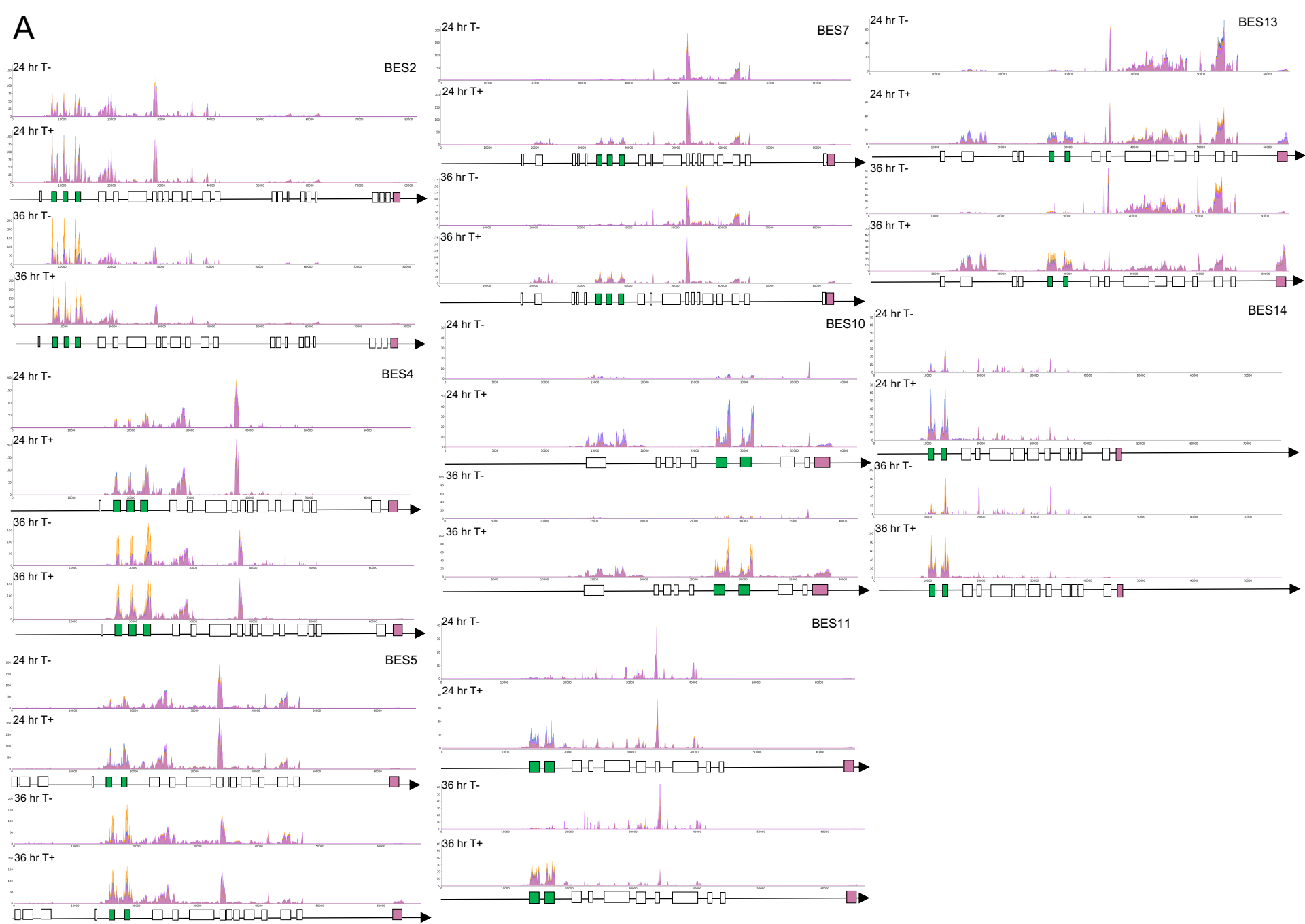

**Figure S3**

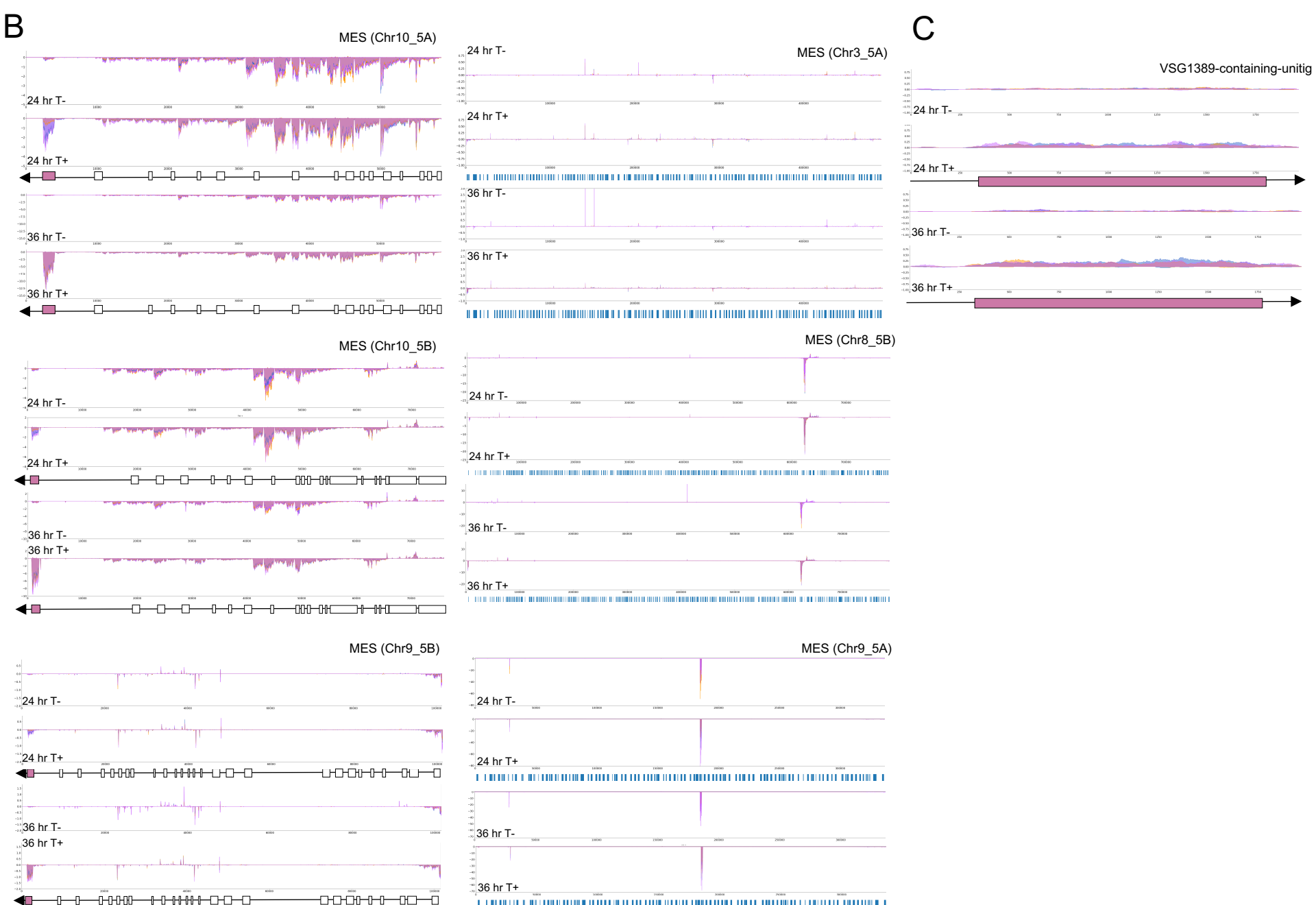

**Figure S3**

Supplement: Data S1. Additional Plots from the RNA-Seq Mapping, Related to Figures 3 and S3 — See Figure S3 for explanation of content. [file mmc6.pdf]
